# Global Transcriptional Analysis Reveals the Complex Relationship between Tea Quality, Leaf Senescence and the Responses to Cold-Drought Combined Stress in *Camellia sinensis*

**DOI:** 10.3389/fpls.2016.01858

**Published:** 2016-12-09

**Authors:** Chao Zheng, Yu Wang, Zhaotang Ding, Lei Zhao

**Affiliations:** Tea Research Institute, Qingdao Agricultural UniversityQingdao, China

**Keywords:** *Camellia sinensis*, cold-drought combined stress, leaf senescence, tea quality, RNA-Seq

## Abstract

In field conditions, especially in arid and semi-arid areas, tea plants are often simultaneously exposed to various abiotic stresses such as cold and drought, which have profound effects on leaf senescence process and tea quality. However, most studies of gene expression in stress responses focus on a single inciting agent, and the confounding effect of multiple stresses on crop quality and leaf senescence remain unearthed. Here, global transcriptome profiles of tea leaves under separately cold and drought stress were compared with their combination using RNA-Seq technology. This revealed that tea plants shared a large overlap in unigenes displayed “similar” (26%) expression pattern and avoid antagonistic responses (lowest level of “prioritized” mode: 0%) to exhibit very congruent responses to co-occurring cold and drought stress; 31.5% differential expressed genes and 38% of the transcriptome changes in response to combined stresses were unpredictable from cold or drought single-case studies. We also identified 319 candidate genes for enhancing plant resistance to combined stress. We then investigated the combined effect of cold and drought on tea quality and leaf senescence. Our results showed that drought-induced leaf senescence were severely delayed by (i) modulation of a number of senescence-associated genes and cold responsive genes, (ii) enhancement of antioxidant capacity, (iii) attenuation of lipid degradation, (iv) maintenance of cell wall and photosynthetic system, (v) alteration of senescence-induced sugar effect/sensitivity, as well as (vi) regulation of secondary metabolism pathways that significantly influence the quality of tea during combined stress. Therefore, care should be taken when utilizing a set of stresses to try and maximize leaf longevity and tea quality.

## Introduction

Tea plant (*Camellia sinensis* (L.) O. Kuntze), an evergreen woody plant, contains various secondary metabolites that are potentially beneficial to human health, and its young leaves are processed to prepare “tea.” Just like other plants, being sessile in nature, tea plants are continuously challenged by a wide variety of environmental stresses, such as drought and low temperature (Zheng et al., 2015; Liu et al., 2016). Every year, substantial loss of tea production and degradation of tea quality due to leaf senescence can result from a particular or often a combination of different stress conditions (Liu et al., 2015; Wang et al., 2016). In field conditions, cold stress often occurs in conjunction with drought stress, and this combination has been recognized as realistic threats faced by plants in the winter of semi-arid or drought-stricken areas (Su et al., 2015). Previously, a few notable studies demonstrated that stress interactions provoke altogether different signaling events and transcriptional responses that were unpredictable or not seen under either of the single stresses (Rasmussen et al., 2013; Pandey et al., 2015). In a recent study, the physiological responses were compared between drought-sensitive and drought-tolerant sugarcane cultivars under cold and drought stress in single, and double combination (Sales et al., 2013). The drought-resistant genotype maintained plant growth under cold and drought either alone or combined via enhancing the antioxidant capacity, which was not observed in the sensitive genotype. In addition, a rapid recovery of photosynthesis after combined stress was observed to be associated with high APX and SOD activities in drought-resistant plants. These findings support the role of the antioxidant mechanisms to be an essential strategy to guard plants against stress combinations (Pandey et al., 2015). Although cold and drought stress have been extensively studied in tea plants, little is known about how their combination impacts plants on a whole-genome basis (Wang et al., 2013, 2016; Zheng et al., 2015; Liu et al., 2016). In recent years, stress-induced premature senescence has become a major threat to crop yield worldwide and considered an important field of research (Schippers et al., 2015). Transcriptome studies have revealed the existence of considerable cross-talk between leaf senescence and drought stress responses (Munné-Bosch and Alegre, 2004). Leaf yellowing (i.e., chlorophyll degradation along with carotenoid accumulation) and specific changes in phytohormone balance (e.g., increase in ABA and decrease in cytokinins), metabolism (e.g., lipid peroxidation, protein degradation, and ROS detoxification) and gene expression (e.g., up-regulation of *LEA* and *DREB2A*) occur gradually during leaf senescence in drought-stressed plants (Munné-Bosch and Alegre, 2004; Schippers et al., 2015). Leaf senescence is under strict genetic control and represents an important evolutionary trait that enables plants to regulate nutrient use efficiency and phenotypic plasticity of growth under drought stress (Schippers et al., 2015). In several plant species, however, the stay-green phenotype is observed to exhibit a better drought resistance. For example, (Rivero et al., 2007) engineer drought tolerance by delaying drought-induced senescence via up-regulation of isopentenyl transferase gene (*IPT*) involved in cytokinin biosynthesis in tobacco. Low temperature, on the other hand, can result in delayed senescence and extends plant longevity. Overexpression of CBF2 and CBF3 genes that involved in cold acclimation, remarkably delays the leaf senescence in *Arabidopsis* (Sharabi-Schwager et al., 2010b). CBFs seem to delay the onset of leaf senescence via regulating their target genes, such as *RD29* and *COR15* to control senescence-associated genes (*SAGs*) (e.g., *ANAC019* and *ANAC055*) (Yang et al., 2011; Koyama, 2014). Agronomically, stress-induced leaf senescence is immensely important, since the efficiency and timing of nutrient remobilization in crops are not only linked to yield, but strongly influence the nutritional quality of our food (Schippers et al., 2015). Currently, little is known about the molecular mechanism of stress-induced leaf senescence in *C. sinensis* and how leaf senescence is delayed by low temperature has not been investigated at the transcriptome level so far.

During stress and in senescing leaves, a wide variety of secondary metabolites (e.g., flavonoids, volatile compounds, and theanine) is synthesized from primary metabolites (Ramakrishna and Ravishankar, 2011; Schippers et al., 2015). They may act as signal molecules or elicitors to confer protection against abiotic factors in plants. For example, exposure to drought or cold stress can cause inactivation of enzymes and lipid peroxidation due to the over-production of ROS. To minimize damage, cells produce antioxidants such as flavonoids to scavenge excessive ROS (Wu et al., 2014). These secondary metabolites also contribute to the specific colors, odors and tastes in plants, and are unique sources for bio-pesticides, food additives, fragrances and pharmaceuticals. Tea quality is defined by color, taste, and aroma. While volatile compounds are fundamental for tea odor and aroma phenolic compounds and free amino acids are responsible for the color and the taste (Yang et al., 2013). If one considers the economic implications of tea, it is not surprising that the metabolic pathways and enzymes involved in the formation of tea flavonoids, theanine, and volatile compounds have attracted the attention of many researchers. Recently, (Li et al., 2015) analyzed the gene expression profiles of 13 different tissue samples from various organs and developmental stages, which further elucidated the gene network responsible for the regulation of flavonoid, caffeine and theanine biosynthetic pathways in tea plants; (Wang et al., 2016) analyzed secondary metabolism changes in tea leaves during short-term drought stress using RNA-Seq technology. They revealed drought stress resulted in a significant increase in the total flavonoids and a decrease in the free amino acids and total polyphenols. As mentioned above, the response to co-occurring stress does not reflect a simple merge of the single stress responses, but activate an entirely new program of gene expression. Therefore, it cannot be assumed that the concentrations of tea secondary metabolites that accumulate due to drought or cold stress would be additive if the two stressors occurred together.

To take a further step toward understanding plant responses to complex environmental conditions, we examined the global transcriptome profiles of tea leaves under single cold and drought stress, as well as their combination using RNA-Seq technology. Our analysis showed that plants exposed to combined cold and drought stress experience a totally different stress and trigger some entirely new molecular events. Plant response to co-occurring cold and drought stress consists of both unique and shared adaptation strategies that ensures best utilization of limited energy resources. Furthermore, the confounding effect of cold and drought stress on tea quality and leaf senescence was also investigated. This permitted us to shed a new light on the crucial relationship between crop quality, plant longevity, and stress tolerance.

## Materials and methods

### Plant materials and stress treatments

**Figure 1:**
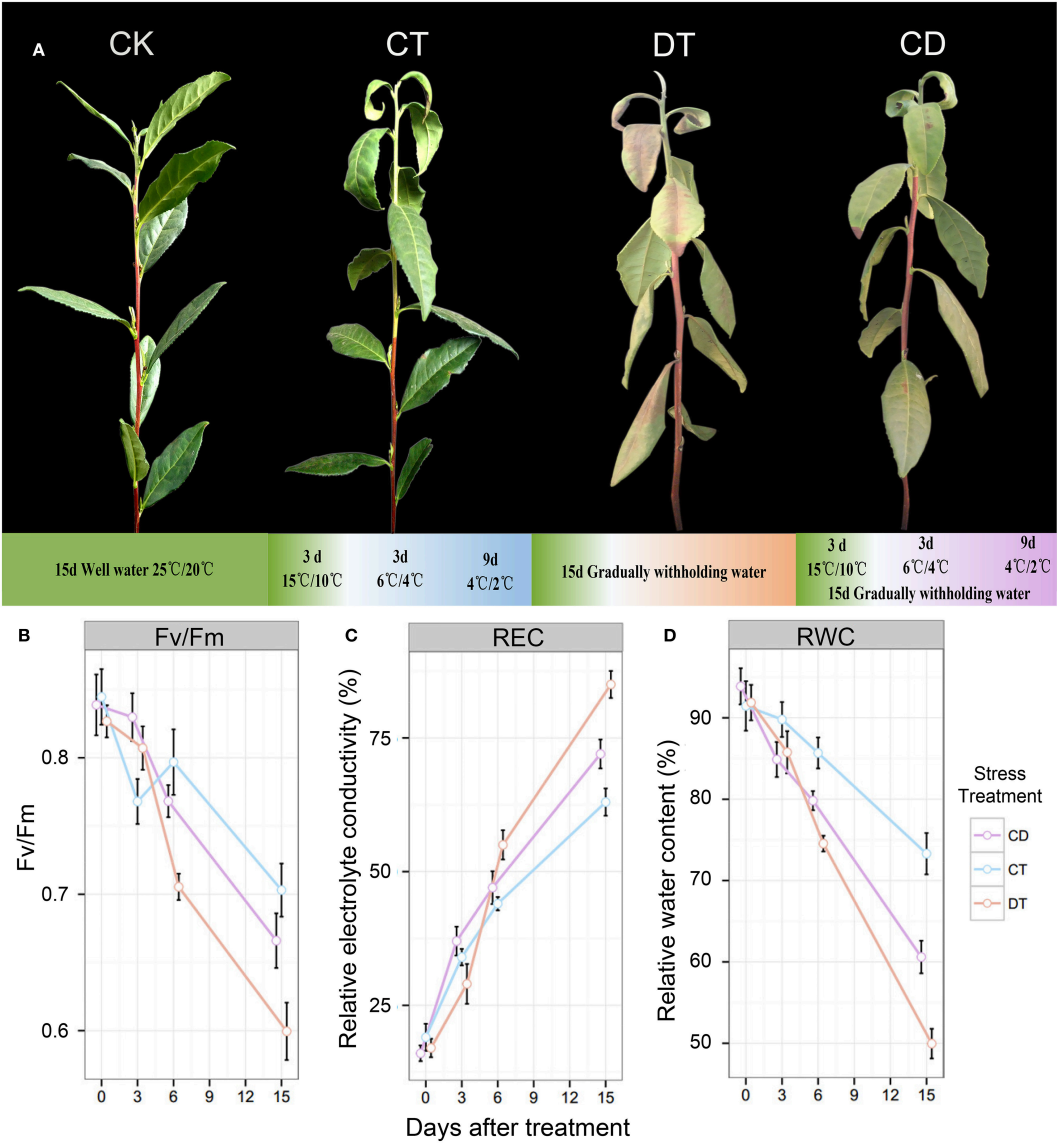
**The phenotypes and physiological analyses of tea plants under CT, DT and CD. (A)** The phenotypes of control (CK) and plants treated with gradually cold (CT) and drought (DT) stress individually or together (CD) after 15 days. **(B)** Leaf maximum photochemical quantum yield of PS II (*Fv/Fm*), **(C)** leaf relative electrolyte conductivity (REC), and **(D)** relative water content (RWC) were determined at six time-points (0, 3, 6, 9, 12 and 15 days) during CT, DT, and CD.

*Camellia sinensis* (L.) O. Kuntze cv. “*Yingshuang*,” an improved cultivar derived from the cross of two major Chinese cultivars, *C. sinensis* var. *sinensis* cv. “*Fuding Dabaicha*” and *C. sinensis* var. *assamica* cv. “*Yunnan Dayecha*,” was used as plant material. It has been identified to possess high tolerance to both cold and drought stress (Wang et al., 2014; Zhou et al., 2014). Two-year-old tea plants were culture-grown under a 12-h light (25°C)/12-h dark (20°C) photoperiod with 1800 Lx photos m^−2^·s^−2^ light intensity and 75% humidity in growth chamber for 2 weeks. For drought stress, plants were treated by gradually withholding water for 15 days. For cold stress, plants were exposed to decreasing temperatures of acclimation (15/10°C) for 3 days and chilling (6/4°C for 3 days and 4/2°C for 9 days) for 12 days. For combined CD stress, plants were simultaneously treated by gradually withholding water, and decreasing temperatures of acclimation and chilling for 15 days (Figure 1A).

### Physiological experiments and RNA extraction

For physiological experiments, more than five plants were harvested and pooled for each treatment group at 0, 3, 6, 9, 12, and 15 days, and the collection was repeated three times as biological replicates. Third leaf was subjected to a FluorCam 700 mf (Photon Systems instruments, Brno, Czech Republic) on the *Fo, Fm*, and Kautsky effect setting to obtain maximum photochemical quantum yield of PS II (*Fv/Fm* = (*Fm* − *Fo*)/*Fm*). Fourth and 5th leaf was used for estimating relative electrolyte leakage (REL) and relative water content (RWC), respectively. REL and RWC were determined as described previously (Su et al., 2015). The total RNA was isolated from the 3rd leaf for each treatment group at 15 days as described previously (Zheng et al., 2015). RNA quality and quantity was determined using 1% agarose gel electrophoresis, NanoDrop Photometer Spectrophotometer (IMPLEN, Westlake Village, CA, USA), Qubit RNA Assay Kit in Qubit 2.0 Flurometer (Life Technologies, Carlsblad, CA, USA), and RNA Nano 6000 Assay Kit of the Bioanalyser 2100 system (Agilent Technologies, Santa Clara, CA, USA). Only the RNA samples with RIN (RNA integrity number) more than 8.0, 260/230 ratio between 2.0 to 2.5 and 260/280 ratio between 1.8 and 2.0, were used for sequencing.

### Illumina sequencing and *de novo* assembly

For transcriptome library construction, 1.5 μg of total RNA per sample was used for the RNA sample preparations. The library for sequencing was generated using NEBNext Ultra RNA Library Prep Kit for Illumina (NEB, USA). After cluster generation, the library preparations were sequenced on an Illumina Hiseq 4000 platform at Novogene (Novogene, Beijing, China), using TruSeq PE Cluster Kit v3-cBot-HS (Illumina), 150 bp paired-end reads were generated from transcriptome sequencing. ~563 million high quality RNA-Seq reads were pooled from Illumina sequencing of each of the 12 samples (three biological replicates for CT, DT, and CD) and were then assembled into unigenes using Trinity (Release 2012-04-27; Grabherr et al., 2011).

### Sequence annotation

Gene function was annotated based on the following databases: Nr (NCBI non-redundant protein sequences); Swiss-prot (A manually annotated and reviewed protein sequence database); Pfam (Protein family); Nt (NCBI non-redundant nucleotide sequences); GO (Gene Ontology); KO (KEGG Ortholog database); KOG (euKaryotic Ortholog Groups). All the unigenes were searched against Nr, Nt, Swiss-prot, KO and KOG databases using the BLAST algorithm (*E* < 1E-5). On the basis of Swiss-prot BLAST results, the Blast2GO program was used to perform GO functional classification (Götz et al., 2008). When a unigene could not be found in any of the above databases, ESTScan was used to decipher its coding regions and sequence orientation orders (Iseli et al., 1999).

### Differential gene expression, GO and KEGG enrichment analysis

Differential expression analysis was performed using the DESeq R package (1.10.1) (Anders and Huber, 2010). The resulting *P*-values were adjusted using the Benjamini and Hochberg's method. Genes with an adjusted *P* < 0.05 found by DESeq were assigned as differentially expressed. GO enrichment analysis of the differential expressed genes (DEGs) was implemented by the GOseq R packages based Wallenius non-central hyper-geometric distribution (Young et al., 2010). KEGG pathway enrichment analysis was done using KOBAS software (KOBAS, Surrey, UK; Mao et al., 2005).

### qRT-PCR validation for DEGs

To verify RNA-seq results, 16 DEGs were selected for qRT-PCR test. qRT-PCR was performed using SYBR Premix Ex Taq II kit (Takara) and run on LightCycler 480 Real-Time PCR System (Roche Applied Science) under the following parameters: 95°C for 30 s, 40 cycles at 95°C for 5 s, 60°C for 30 s. Triplicates of each reaction were performed, and GAPDH sequence was used as endogenous control. CT values obtained through qRT-PCR were analyzed using 2^−ΔΔCT^ method to calculate relative fold change values (Livak and Schmittgen, 2001). The details of the primers used in qRT-PCR are given in Supplementary Table 12.

### Accession code

RNA-seq read data were deposited to the NCBI Sequence Read Archive (NCBI SRA) under accession number SRP091321.

## Results

### Physiological characterization of tea plants subjected to cold and drought in single and double combination

To compare the impact of single and combined stress on physiological and transcriptional plant responses, tea plants were exposed to cold, drought, and their combination. The optimal experimental conditions for single stress were defined first and then combined. Mild conditions were chosen to mimic long-term stresses that naturally occur in the field compared with the drought or cold shock used in comparable studies. As illustrated in Figure 1A, drought treatment (DT) was applied by gradually withholding water and lasted 15 days to induce leaf senescence. For cold treatment (CT), plants were exposed to decreased temperatures of acclimation (15/10°C) for 3 days and chilling (6/4°C for 3 days and 4/2°C for 9 days) for 12 days For combined stress treatments (CD), we applied cold and drought stress simultaneously for 15 days. Well-watered plants under ambient temperatures (25°C day/20°C night) were used as a control (CK). After 15 days of stress treatment, a number of curly yellow and reddish-brown leaves were observed in DT, while for CT, only some wilting and yellow patches were observed (mainly 1st–3rd leaf). As for CD stressed plants, leaf senescence was remarkably delayed, and drought did not induce the severe senescence response (Figure 1A). As a starting point for the analysis, we determined relative electrolyte conductivity (REC), relative water content (RWC), and leaf maximum photochemical quantum yield of PS II (*Fv/Fm*) of different treatment groups at six time-points (0, 3, 6, 9, 12, and 15 days) (Figures 1B–D). It was expected that both cold and drought stresses may cause cell dehydration and over-accumulation of ROS, resulting in damage to membrane and photosynthesis system at the cellular level. At the time of harvest, a significant reduction in RWC and *Fv/Fm*, and an increase in EL were found for CT and CD, which was emphasized even more on DT (Figures 1B–D). These results are in line with our observations of morphological changes, indicating the potential positive interactions between cold acclimation and drought stress.

### *De novo* assembly of transcriptome and functional annotation of unigenes

To compare the molecular events in control and plants treated with cold and drought stress individually or together, 12 RNA-Seq libraries of three biological replicates for CT, DT, CD, and CK were prepared and then paired-end sequenced. For each sample, sequence data ranging from 5.87 to 8.84 Gb were generated (Supplementary Table 1). After discarding the low-quality raw reads, a total of 562,692,884 high-quality reads were used for a *de novo* assembly of the leaf transcriptome. In total, 268,889 transcripts, and 170,102 non-redundant unigenes with a N50 of 894 bp and an average length of 615 bp were achieved (Supplementary Table 2). We then searched these unigenes against seven public databases, including Nr, Nt, KOG, GO, KEGG, Pfam, and Swiss-prot for identifying homologous sequences. A total of 66,433 (39.05%) unigenes were annotated in at least one databases, and the detailed annotation information and the distributions of functional categories in GO and KEGG databases were listed and displayed in Supplementary Table 3 and Supplementary Figure 1, respectively.

### Identification and clustering analysis of DEGs under cold and drought in single and double combination

**Figure 2:**
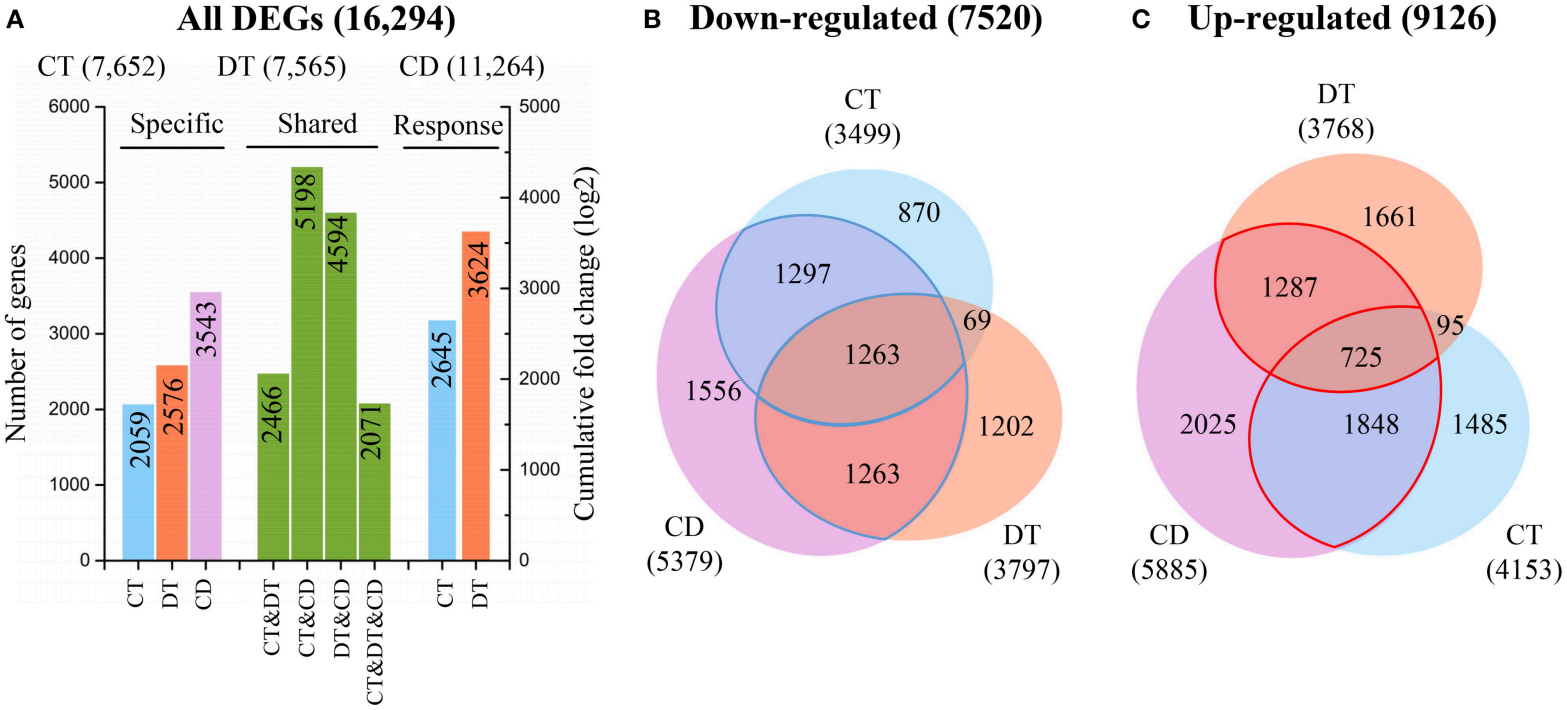
**Relationships between transcriptome responses during CT, DT, and CD. (A)** The histogram showing the number of common and specific DEGs during CT, DT, and CD (left), and the cumulative log-fold changes of 1000 most significantly CT- and DT-responding unigenes in CD (right), respectively. Venn diagram of genes **(B)** down-regulated or **(C)** up-regulated by each stress. The numbers of common and specific DEGs were shown in the overlapping and non-overlapping regions, respectively. The total numbers of up- and down-regulated DEGs were indicated in parentheses.

To examine the molecular differences between single and combined stress responses, the DESeq R package was used to identify significantly DEGs from each stress group and control (Anders and Huber, 2010). The number of CT (7652) and DT (7565) responsive genes are nearly the same, while the number of genes responding to CD (11,264) are far greater (Figure 2A). Our analysis also revealed a substantial number of CD-unique DEGs (3543, 31.5%), which were absent from the list of DEGs under CT and DT. In addition, only 2071 genes (12.7% of all DEGs) were modulated in all the three stress conditions, and only 7721 of genes (47.4% of all DEGs) that responded to either CT or DT were activated by CD (Figure 2A). The CD triggered a totally different and higher response compared to each of the different stresses applied individually, suggesting that tea plants may develop some distinct response mechanism when presented with combined CD stress. To test the accuracy of our RNA-Seq data, quantitative reverse transcriptase PCR (qRT-PCR) was performed on 16 DEGs, and we found the data from both were highly consistent (Supplementary Figure 2).

To further investigate the contributions of each single stress to the response to combined stress, we calculated the cumulative log-fold changes of 1000 most significantly CT- and DT-responding unigenes in CD, respectively (Figure 2A). We found a higher contribution of DT (3624) to the response to CD while compared to CT (2645), although the genes commonly modulated in DT and CD (4594) was lower than the share between CT and CD (5198) (Figure 2A). These observations suggested that higher number of stress responsive genes does not always have a greater impact on gene expression under combined stress.

The Venn diagram analysis of DEGs indicated that a great number of genes induced by the single stress were not fully induced by combined stress (Figures 2B,C). Among the 7101 genes that are induced by at least one single stress, only 3860 (54%) are also induced by combined stress (Figure 2C). Furthermore, among the 5964 genes that are repressed by at least one single stress treatment, 3823 (64%) are also repressed by combined stress (Figure 2B). This suggested that tea plants tend to repress the genes should be repressed, but can fail to activate the genes should be activated under combined CD stress.

**Figure 3:**
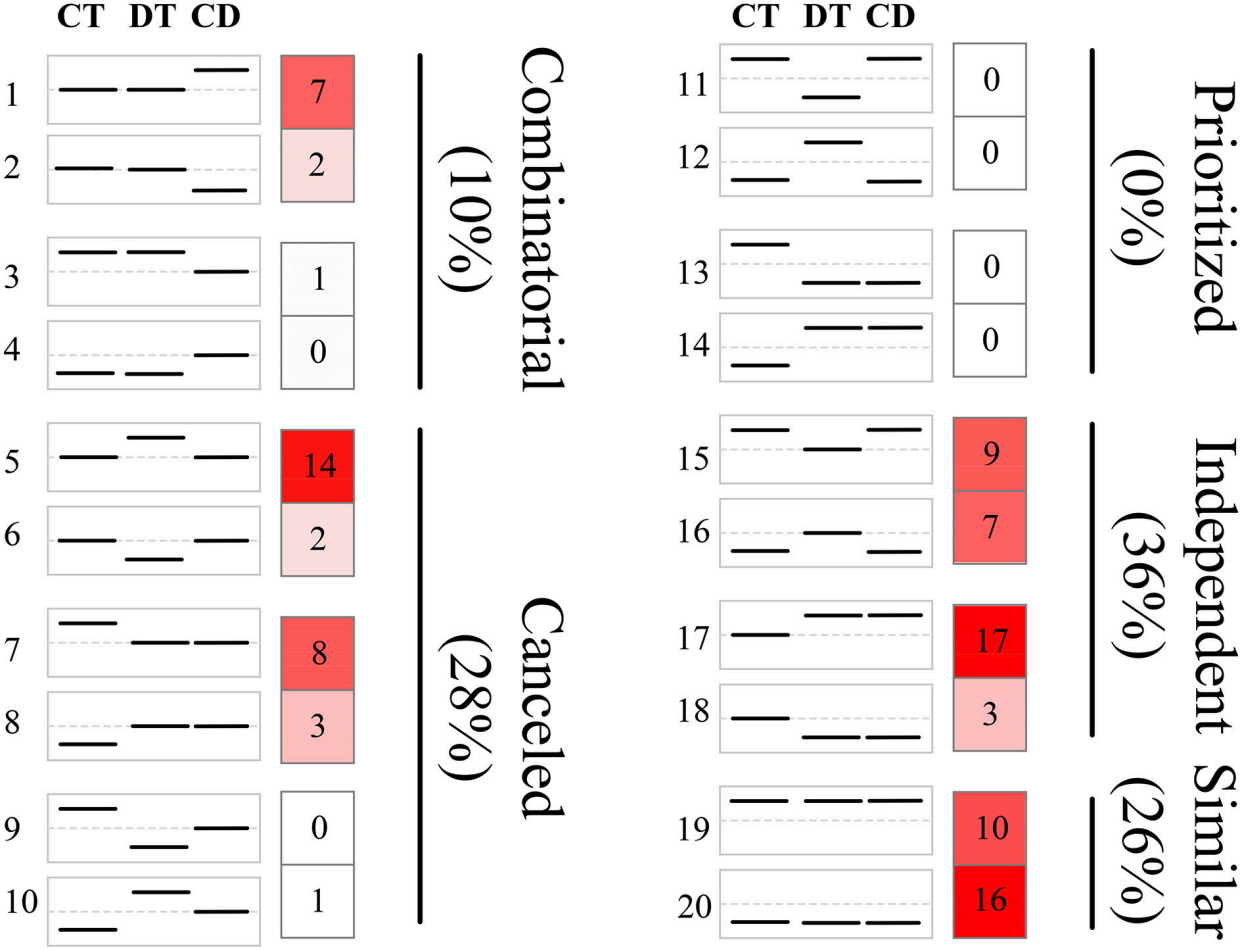
**Clustering of unigenes to predefined expression profiles generating the transcriptional response modes**. The unigene sets were created by the union of the 500 most significant unigenes for CT, DT, and CD. These unigenes were clustered to 20 predefined expression profiles, each categorizing a potential expression change that may occur when combined stresses are applied. The dotted line in the boxes represents unigene expression with no change compared with the control. The value and color in the right boxes represents the percentage of unigenes that correlate with the particular predefined expression profile (red is higher). Combinatorial, similar levels in the CT and DT but a different response to CD; canceled, unigene response to either CT or DT returned to control levels; prioritized, opposing responses to the CT and DT and one stress response prioritized in response to CD; independent, response to only CT or DT and a similar response to CD; similar, similar responses to CT, DT, and CD.

The responses of a given unigene to CT and DT may be antagonistic, agonistic, unrelated, or neutral, and the response to the combined CD stress may be a combination of such responses. To investigate these transcriptional response modes, we clustered the union of the top 500 most significant DEGs from CT, DT, and CD to predefined expression profiles as described by Rasmussen et al. (2013) (Figure 3; Supplementary Table 4), and the transcriptional response mode assignments were stable using the different number of significant unigene sets (i.e., 500, 1000, and 2000 most significant unigenes) (Supplementary Figure 3). The majority of the transcriptional response modes were “independent” (36%), “canceled” (28%), and “similar” (26%), followed by “combinatorial” (10%). Only 6 unigenes (0%) responded in the “prioritized” mode indicating that responses to CD involve relatively few antagonistic interactions between CT and DT responding genes. In addition, 38% of the unigenes were involved in the “prioritized,” “combinatorial,” and canceled” mode, whose expressing patterns under CD cannot be predicted from the CT or DT alone due to the interaction of two kinds of stress responses. The majority of the genes in the “combinatorial” modes were a larger fraction of CD-unique DEGs (9% out of 10%). A large number of DT-induced genes showed a “canceled” mode when combined with CT (14% out of 28%). As for the “independent” modes, the effects of DT may contain a large set of up-regulation unigenes (17% out of 36%) that are not influenced by CT and may solely be associated with DT.

**Figure 4:**
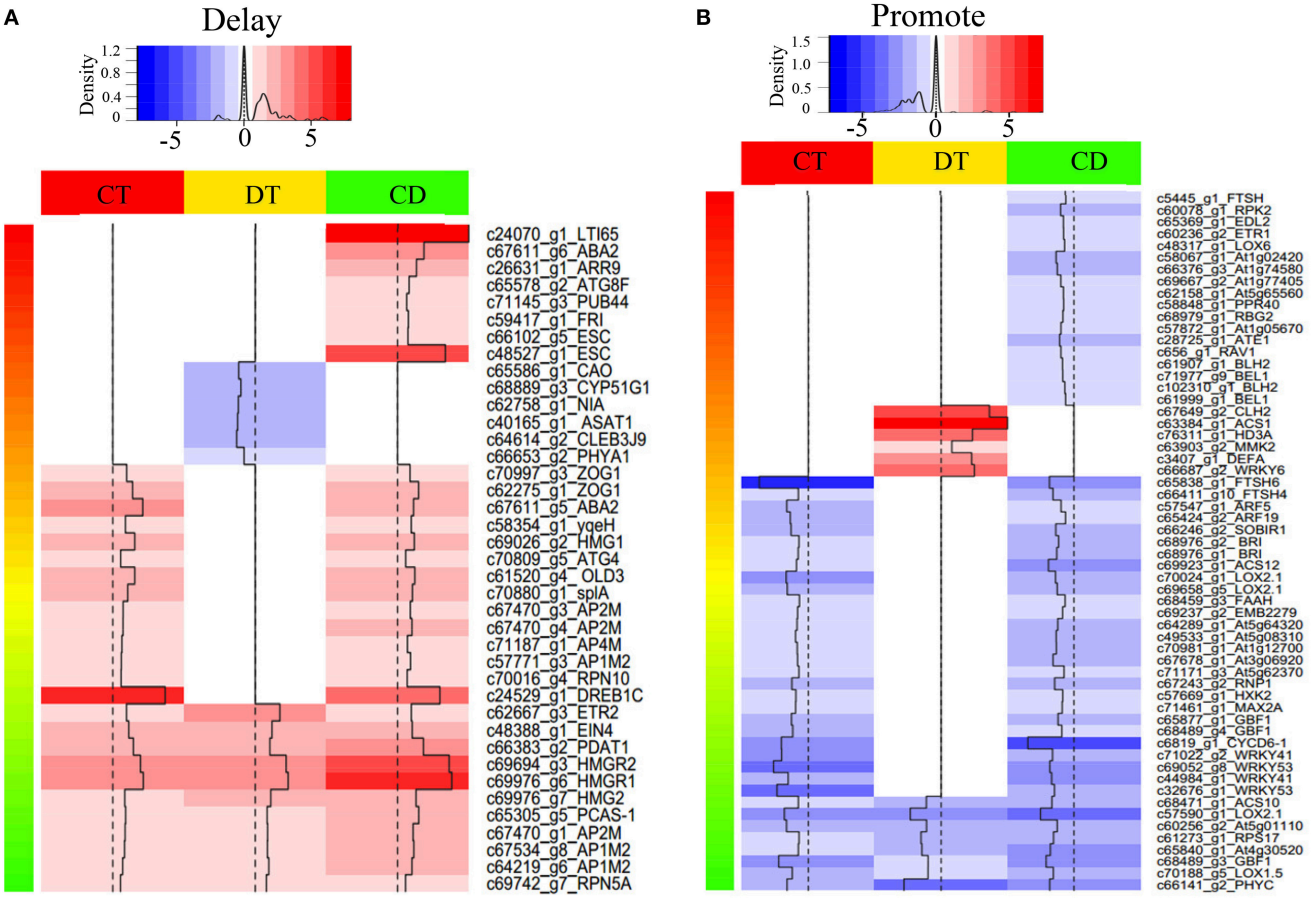
**Heat map of selected differentially expressed *SAGs* with known effect**. Heat map shown the expression level of SAGs with promote **(A)** or delay **(B)** effect in LSD. The selection was based on the clustering analysis results (i.e., mode 1, 2, 5, 6, 9, 10, 11, 12, 15, 16, 19, and 20 in Figure 3). Color indicates fold change of DEGs under CT, DT and CD, as shown in the top.

To assess how low temperature can delay drought-induced senescence, we performed a BLAST search of 16,294 DEGs against 6446 SAG sequences reported on the Leaf Senescence Database (LSD) (Li et al., 2014). The output of the BLAST search was that 6878 candidate unigenes displayed high sequence similarity. Among these candidates, 3538, 3406, and 4756 putative *SAGs* were differentially expressed under CT, DT, and CD, respectively. Based on the clustering analysis results (i.e., mode 1, 2, 5, 6, 9, 10, 11, 12, 15, 16, 19, and 20 in Figure 3), we further selected *SAGs* with known effect (promote or delay) in LSD to mining the key genes that have a potential role in delaying leaf senescence under CD (Figure 4; Supplementary Table 5). Among the delaying subsets (39), the most significantly expressed categories were genes associated with chlorophyll biosynthesis (*CAO*), nutrient recycling (*ATG8F* and *ATG4*), hormone response pathway (e.g., *ABA2, ARR9*, and *EIN4*), lipid metabolism (e.g., *ASAT1, PDAT1* and *HMG1*/*2/R1*/*R2*), stress response (e.g., *LTI65*/*RD29B* and *DREB1C*), signal transduction (e.g., *ESC, PHYA1, FRI*) and protein degradation/modification (e.g., *PUB44, RPN10*/*5A*, and *AP1M2/2M/4M*) (Figure 4A; Supplementary Table 5). As for the promote subsets (59), genes related to carbohydrate metabolism (*HXK2*), chlorophyll degradation (*CLH2* and *FTSH4/5/6*), hormone response pathway (e.g., *BRI1/SR160, ARF5/19, ETR1, ACS1/10/12*, and *LOX2.1/6*), signal transduction (e.g., *GBF1, CYCD6-1, HD3A, PHYC*, and *MMK2*) and transcription regulation (e.g., *RAV1, BEL1, DEFA*, and *WRKY6/41/53*) were overrepresented. Most of these *SAGs* have clear functional connections to senescence programs (Figure 4B; Supplementary Table 5). For example, *DREB1C/CBF2* (c24529_g1), a well-known transcription activator acts to enhance plant frost tolerance, was up-regulated in CT and CD (Heidarvand and Maali Amiri, 2010). This gene has also previously been shown to delays the onset of leaf senescence and extends the life span of the plants (Sharabi-Schwager et al., 2010a). Noteworthy, both the target (*LTI65*/*RD29B*, c24070_g1) and the activator (*CAMTA3*, c71106_g1) of CBF2 can be found significantly induced exclusively in CD. *ARF19* (c65424_g2) encoding a member of the auxin response factor were repressed in CT and CD. This gene is known to function as a transcriptional activator to enhance the senescence and abscission phenotypes (Ellis et al., 2005). *LOX2.1* (c70024_g1 and c69658_g5) involved in JA-mediating leaf senescence was repressed in CT and CD (He et al., 2002). *HMG1* (c69026_g2) was significantly induced in response to CT and CD, and it has been implicated in modulating cell elongation and senescence by promoting triterpene biosynthesis (Suzuki et al., 2004). These results suggested that although DT usually results in leaf senescence, CT can delay the senescence process by manipulating a set of transcription factors (TFs), hormone regulation-related genes and *SAGs* in tea plants.

### Functional annotation of DEGs reveals the unique and shared responses to single and combined stress

**Figure 5:**
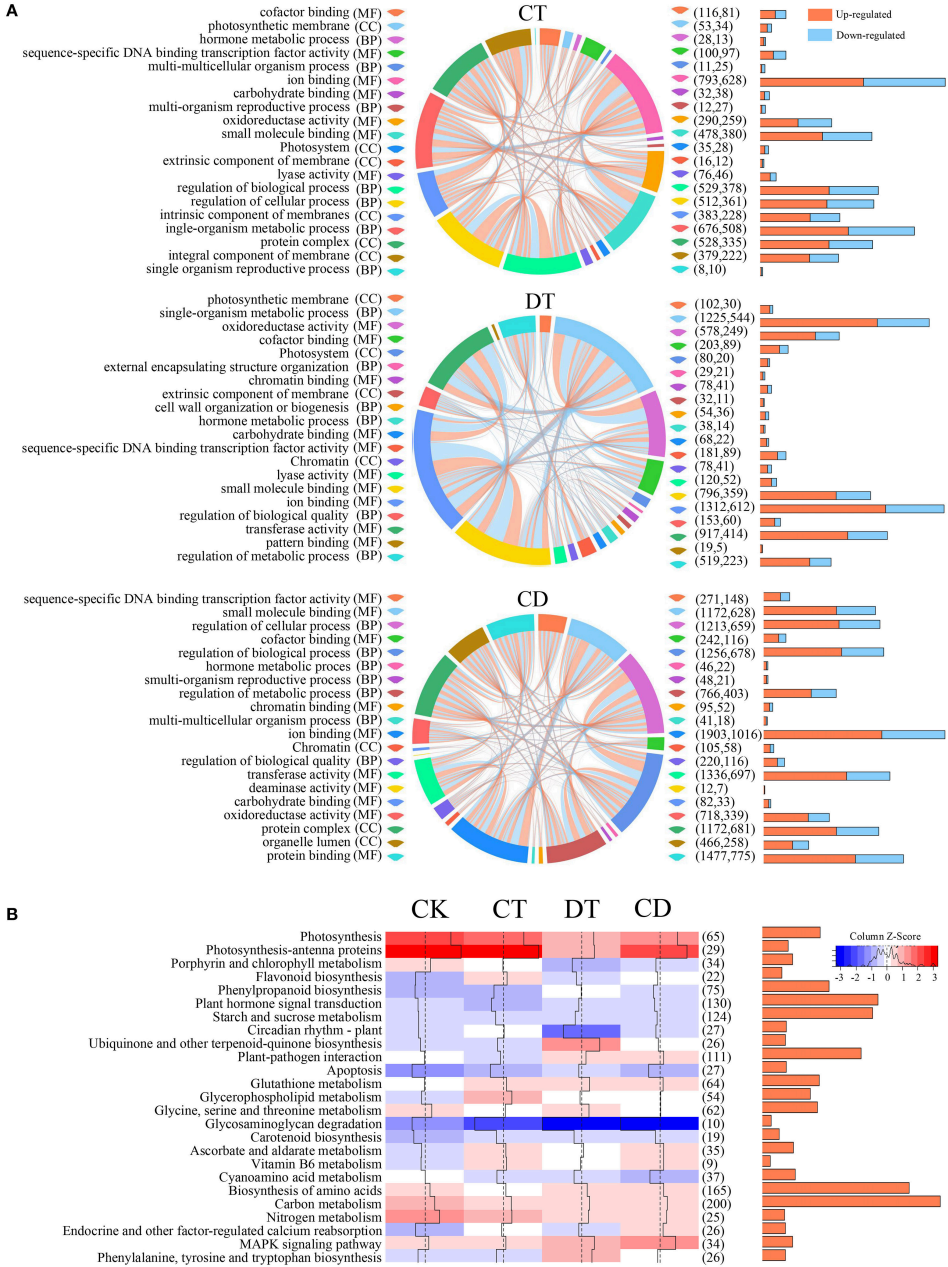
**GO and KEGG pathway analysis of DEGs under CT, DT, and CD. (A)** Multi-series chord graphs for the top 20 level-3 GO terms of each stress treatment. The size of the segments around the circumference of the circle indicate the number of DEGs in specific GO terms. The orange (up-regulated) or blue (down-regulated) lines within the circle indicate the DEGs involved in different biological processes. The numbers of up- and down-regulated DEGs were shown in the right bar graphs and parentheses. **(B)** Heat map showing the overall expression levels of 25 enriched pathways during CT, DT, and CD. The color represents the mean expression values of DEGs within the pathways. The total numbers of DEGs in the pathways were shown in the right bar graphs and parentheses.

To understand the functional differences between CT, DT, and CD responses in *C. sinensis*, we performed KEGG and GO enrichment analysis to explore the relevant pathways and biological functions. A multi-series chord was used to visualize the top 20 level-3 GO terms of each treatment (Figure 5A). The GO terms of CT, DT, and CD were similar and both included terms associated with transcription factor activity, oxidoreductase activity, carbohydrate binding, and hormone metabolism. In spite of the common modulations involved, the three kinds of stressors triggered different responses. For instance, the GO terms relevant to integral/intrinsic component of membrane, cell wall organization or biogenesis and deaminase activity was enriched exclusively in CT, DT, and CD, respectively.

We then performed GO classification analysis for the CD-unique DEGs, and we found functional category “growth” and “cell growth” were significantly up-regulated, while down-regulated terms include “death” and “cell death” (Supplementary Figure 4; Supplementary Table 6). In addition, genes relevant to signaling transduction and stress response were mostly induced among the CD uniquely responsive genes. These results hinted toward prioritized plant responses for growth, signaling and stress responses under combined CD stress. Accordingly, membrane protection (e.g., *FAD3*), cell wall maintaining/remodeling (e.g., *CALS8/10/12, UPTG2, KAM1, GMGT1*, and *EXPA1*), redox (e.g., *GRXS10, PNC2, APX2*/*6 and PER25/48*), and stress response (e.g., *LEA1, HSP70-14/15/16*, and *HSFA4A*) related genes were highly induced among the CD-unique DEGs, whilst chlorophyll degradation (e.g., *FTSH*) related genes were repressed. Meanwhile, calcium (e.g., *CCAMK, CML31*, and *CPK2*), phytohormones (e.g., *IAA30, GA2OX8, PYL8, ARR9*, and *ERF061*/*118*), and MAPK signaling pathway (e.g., *MPK9*/*19/20, NPK1, MEKK1*, and *YDA*), transcription regulation (e.g., *C3H, NAC002, MYB108, WRKY71, BHLH35*, and *VIP1*) and ubiquitin-26S proteasome system (e.g., *XERICO* and *KEG*) were further activated by combined CD stress. For example, the RING-type E3 *XERICO* has been shown to increase ABA levels and improve drought tolerance by inducing the expression of *NCED3* (Ko et al., 2006). In our study, two *XERICO* (c52433_g1 and c44256_g1) were uniquely induced in CD, and two *NCED3* (c63576_g6 and c63576_g7) were induced to a highest level in CD. Gibberellin 2-oxidase 8 (*GA2ox8*) and two-component response regulator ARR9 (*ARR9*), the negative regulators of some bioactive GA and CK signaling respectively, were induced exclusively by CD. Both GA and CK have been known as negative regulators of plant abiotic stress tolerance (Yamaguchi and Kamiya, 2000; Ha et al., 2012). This indicated growth-restraining is also an integral part of plant stress tolerance to promote survival during CD (Achard et al., 2006). Callose has been implicated as an essential component in abiotic and biotic stresses tolerance by reinforcing cell wall (Chen and Kim, 2009). The specifically induction of callose synthase 8/10/12 (*CALS8/10/12*) indicating cell wall thickening maybe one of the strategies used by tea plants to cope with combined CD stress (Supplementary Table 6).

KEGG pathway analysis showed that genes modulated in CT, DT, and CD were associated with similar pathways viz., MAPK signaling, photosynthesis, antioxidant, carbon and nitrogen metabolism, starch and sucrose metabolism, and secondary metabolites biosynthesis (Figure 5B). Pathways related to antioxidant protection (“carotenoid biosynthesis,” “flavonoid biosynthesis,” “glutathione metabolism,” and “ascorbate and aldarate metabolism”) were significantly up-regulated, whereas photosynthesis-associated pathways (“photosynthesis,” “photosynthesis-antenna proteins,” and “porphyrin and chlorophyll metabolism”) were down-represented under both single and combined stresses. These observations suggested that the suppression of photosynthesis and the activation of antioxidant system may be considered as common stress responses, which could have some positive functions such as the protection from the photodamage during stress. Apart from these shared stress responses, the combined stress invoked different pathways. For instance, CD-unique DEGs mapped to oxidative phosphorylation, pentose phosphate pathway, fructose, and mannose metabolism, galactose metabolism, alanine, aspartate, and glutamate metabolism, fatty acid metabolism, terpenoids metabolism, zeatin, folate, flavone and flavonol biosynthesis, and various antioxidative pathways, such as peroxisome, glutathione, and ascorbate and aldarate metabolism (Supplementary Table 7). In addition, we observed a more pronounced reduction in photosynthesis-related pathways, enhanced ROS production (up-regulation of respiratory burst oxidase homolog proteins (*Rboh*) and peroxisomal-(S)-2-hydroxy-acid oxidase (*GLO*); Supplementary Table 8), and enriched “apoptosis” pathway in DT, which indicating the occurrence of drought-induced leaf senescence (Figure 5B). We then detailed investigated the “starch and sucrose metabolism” pathway, and we found most starch degradation related genes (e.g., Alpha-amylase 1/1.1, *AMY1/1.1*; Beta-amylase 1/2/3/7, *BAM1/2/3/7*) were up-regulated, while most genes involved in starch synthase (e.g., Glucose-6-phosphate isomerase 1, *PGI1*; Soluble starch synthase 1, *SSI1*; Granule-bound starch synthase 2, *SS2*; Glucose-1-phosphate adenylyltransferase large subunit 1, *ADG2*; and Glucose-1-phosphate adenylyltransferase large subunit, *AGPS1/3*) were repressed under all stress conditions. In addition, sucrose synthesis related genes (e.g., sucrose-phosphate synthase 2/3/4, *SPS2/3/4*) were specifically up-regulated in CT, while genes associated with sucrose degradation (e.g., Acid beta-fructofuranosidase, *TIV1* and Sucrose synthase 2/3, *SUS2/3*) were highly induced in DT while compared to CT and CD. Several key enzymatic genes related to cell wall degradation, loosing and modification (e.g., pectinesterase 2, *PECS-2.1*; and pectinesterase/pectinesterase inhibitor 40, *PME40*) were significantly induced during DT, which was otherwise repressed upon CT and CD. In contrast, enzymatic genes which are essential for the cell wall biosynthesis, such as galacturonosyltransferase 1/10/11 (*GAUT1/10/11*), UDP-glucuronate 4-epimerase 3 (*GAE3*), were specifically up-regulated under CT and CD. Further, two UDP-glucose 6-dehydrogenase genes (*UGD2/3*), which are involved in diverting UDP-Glucose to cell wall biosynthesis, increased abundance in CD exclusively (Supplementary Table 8).

### Identifying candidate genes for enhancing plant resistance to combined cold and drought stress

**Figure 6:**
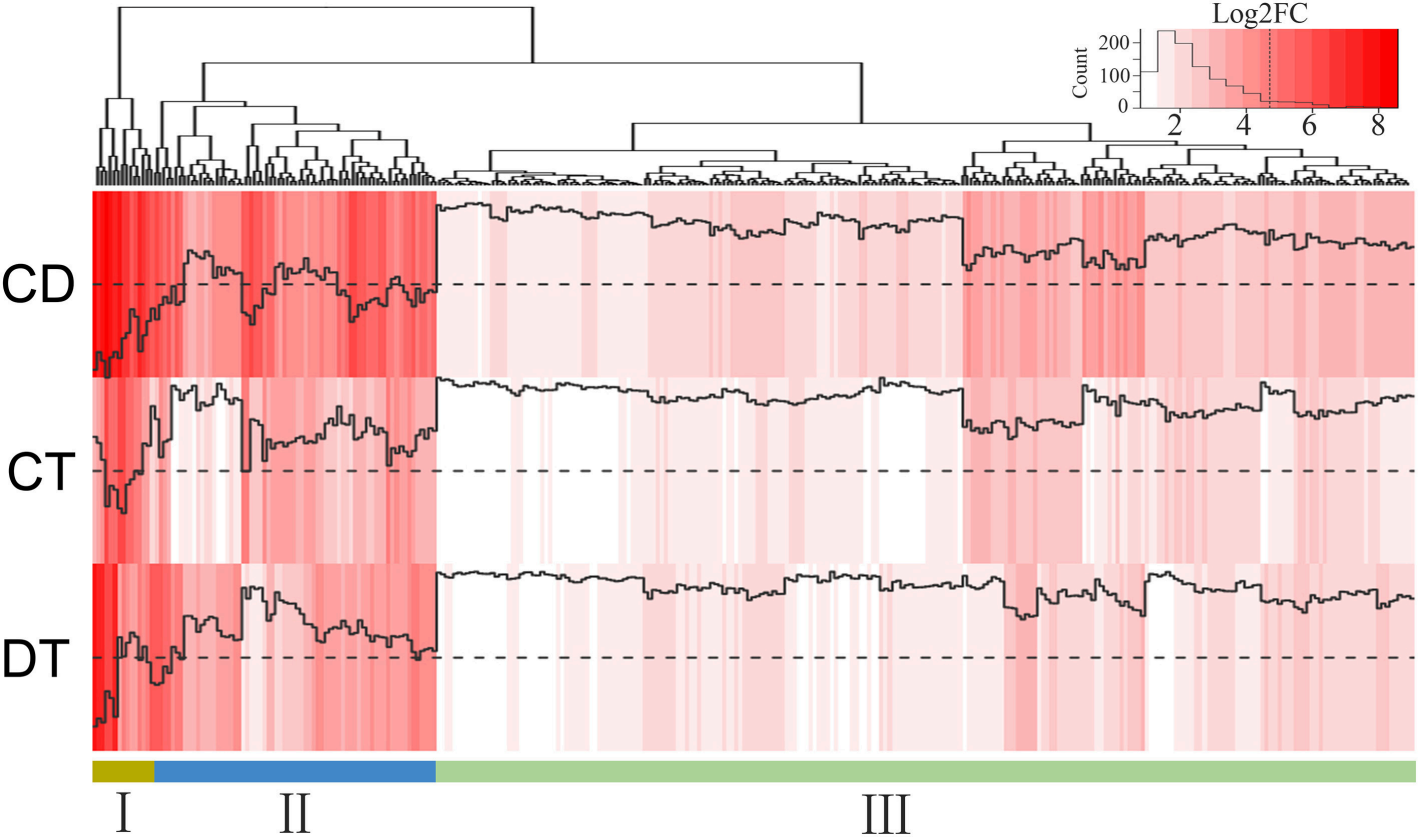
**Heat map of 319 co-up-regulated DEGs that showed enhanced expression under CD compared with CT and DT**. Color indicates fold change of DEGs under CT, DT and CD, as shown in the top.

During a combined stress situation, resource constraints dictate that the plant may be able to induce only the most effective genes. To identify the key genes for producing plants resistant to combined CD stresses, we calculated the fold changes between combined stress and each single stress (i.e., CD/CT and CD/DT) for the 725 co-up-regulated genes, and 319 genes tended to show more enhanced expression in combined stress condition than in both single stresses were retained (Figure 6; Supplementary Table 9). We found these genes were associated with TFs (e.g., *WRKY48, MYB39/306, HSFA8, ERF110/053*, and *DREB2A/C*), detoxification (e.g., *CYP94A2/22*), cell wall remodeling (e.g., *EXPA1 and XTH23*), ubiquitin mediated proteolysis (e.g., *UBC4/32/38, COP1, and ATL42*), hormone signal transduction (e.g., *PLD1, IAA17*, and *PP2C3/AIP1*), terpenoids metabolism (e.g., *HMGR1/2*), carbohydrate metabolism (e.g., *SUC2/7*, and *BAM3*) and stress tolerance (e.g., *LEA5/14-A*). They could be further classified into three subgroups. We observed a higher expression in Group I and II, which are more likely candidates to be involved in plant adaptations to combined CD stress. For example, *SUT2/7* (Sucrose Transporter 2/7) in the Group I, encoding the sucrose transporters responsible for loading of sucrose in source leaves for export to sinks, have shown to be induced by low temperature and osmotic stress via ABA signaling pathway (Gong et al., 2015). Many genes among the Group II (e.g., *NAC7/19, DREB2A/C, LEA5/14-A, NCED3, MYB39/306, and ERF53*) have been known to impart abiotic stress resistance to plants and cope with multiple stresses via ABA-dependent and ABA-independent pathways (Yoshida et al., 2014). DREB2A and DREB2C are key TFs involved in the signal transduction network that control the plant's response to dehydration and cold stress. The DREB2C overexpression lines conferred freezing and heat tolerance to plants, and it was previously reported to interacts with ABF2, a bZip protein regulating ABA-responsive gene expression, and its overexpression affects ABA sensitivity (Lee et al., 2010). LEA proteins may have protective functions during cellular dehydration through mechanisms such as hydration buffering, ion trapping, antioxidant protection, stabilization of sensitive enzymes and membrane protection (Tunnacliffe and Wise, 2007). Overexpressing the *ZmDREB2A* showed upregulation of 44 genes belonging to LEA, heat shock, detoxification proteins and enzymes involved in metabolism, etc. (Qin et al., 2007). However, the overexpression of *DREB2A* also adversely affects plant growth (Sakuma et al., 2006). Intriguingly, gene encoding Growth-regulating factor 7 (*GRF7*, c59210_g1), an inhibitor of DREB2A was highly induced in CT and CD (Kim et al., 2012). This may indicate that tea plants tend to keep up with the trade-off between increased stress tolerance and adverse effects to plant growth in CT and CD. In addition, we found 36.7% (117) of candidate genes has no annotation results on Swiss-prot database, which may be the tea plant-specific genes that response to combined CD stress. Thus, the manipulation of these candidate genes, or their regulators, may represent an effective strategy for the genetic engineering of plant varieties resistant to concurrent cold and drought stresses.

### The effect of the single and combined stresses on secondary metabolite biosynthetic pathways in *C. sinensis*

**Figure 7:**
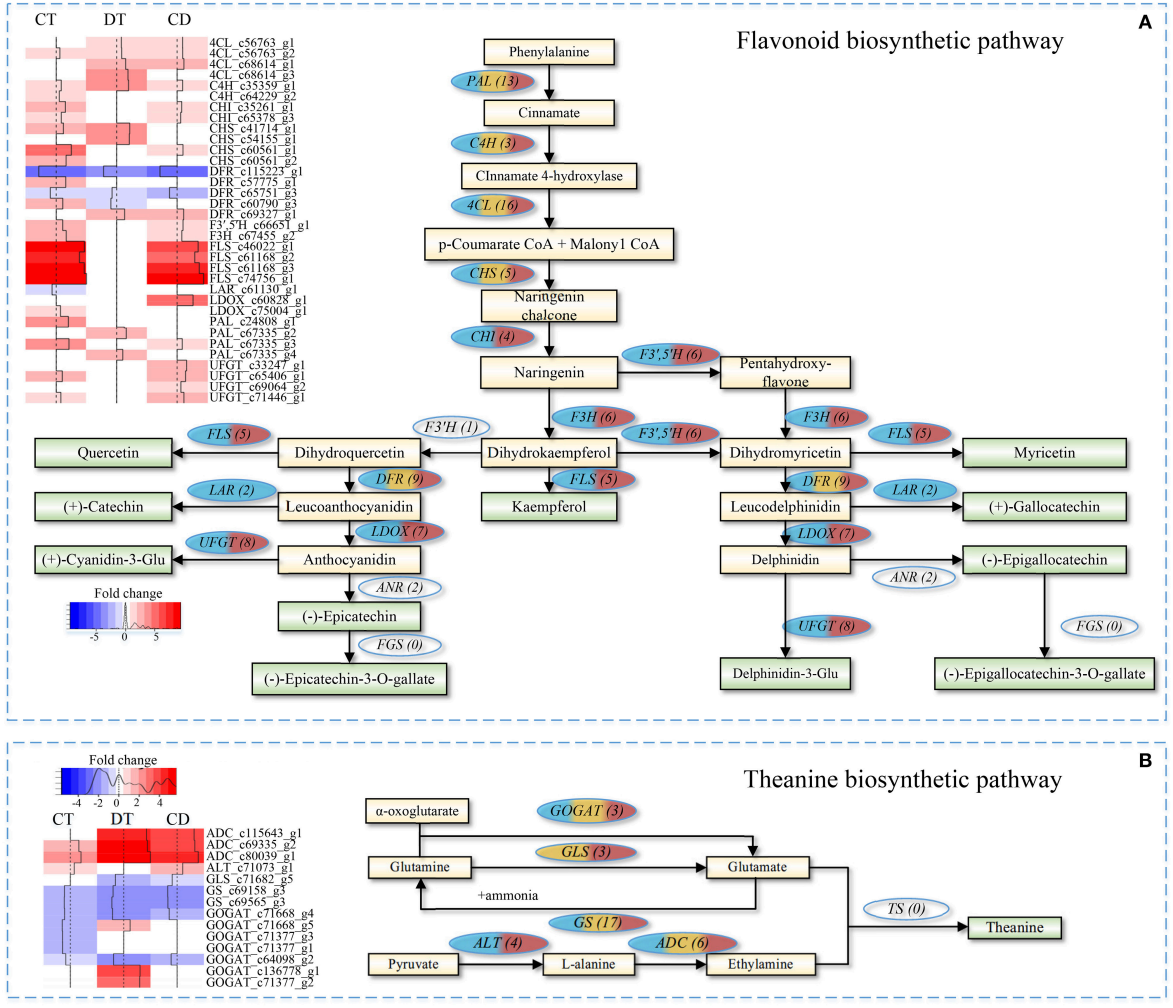
**Flavonoid and theanine biosynthetic pathway in *C. sinensis* under CT, DT, and CD**. Heat maps on the left show fold change of the DEGs involved in **(A)** flavonoid biosynthetic pathway and **(B)** theanine biosynthetic pathway under CT, DT, and CD. The numbers in the brackets following each gene name indicate the number of corresponding unigenes identified in transcriptome. The color in the ellipses represents the gene was regulated under particular stress treatment (blue indicates CT; yellow indicates DT; red indicates CD). Full names of enzymatic genes are expanded in Supplementary Tables 10, 13.

The quality of tea product largely depends on the metabolic profiles of fresh leaf. We focused on pathways involved in volatile compounds, flavonoids and theanine biosynthesis for additional analyses. As shown in Figure 7A, majority unigenes involved in the flavonoid biosynthetic pathway were significantly up-regulated during CT, DT and CD (Supplementary Table 10). Among the pathway, several key enzymatic genes, such as chalcone isomerase (*CHI*), flavonoid 3′,5′-hydroxylase (*F3*′,*5*′*H*), flavonol synthase (*FLS*), flavanone 3-hydroxylase (*F3H*), leucoanthocyanidin oxidase (*LDOX*), and UDP-glucose flavonoid 3-O-glucosyltransferase (*UFGT*) genes, were specifically induced by CT, and CD, suggesting the formation of many types of flavonoids, such as kaempferol, quercetin, and cyaniding during CT and CD. Moreover, leucoanthocyanidin reductase (*LAR*) gene that involved in biosynthesis of catechin and gallocatechin was uniquely repressed by CT. These results suggested that CT possibly play a role in anthocyanidin accumulation but negatively influence the biosynthesis of catechin and gallocatechin in tea plants.

As for theanine pathway (Figure 7B), most of these theanine pathway genes were found in our dataset, except for theanine synthetase (TS) gene. Among the theanine biosynthetic pathway, two glutamine synthetase (GS) genes were down-regulated by all stress conditions, and glutaminase (GLS) gene was down-regulated by DT and CD. Alanine aminotransferase (ALT) converts pyruvate to alanine, and its encoding gene was up-regulated in CT and CD. Glutamate synthase (GOGAT) generates two molecules of glutamate via transferring the amide group from glutamine to 2-oxoglutarate. Three GOGAT genes were up-regulated in DT, while all of them were either down-regulated or uninfluenced in CT and CD. The substrate ethylamine is catalyzed from L-alanine by arginine decarboxylase (ADC). Three ADC genes were significantly up-regulated by all stress conditions, especially in DT and CD (Supplementary Table 10).

The major tea plant volatiles are derived from either the shikimate and terpenoid pathways, or by oxidation of carotenoids and fatty acids (Yang et al., 2013). Our results showed that several enzyme genes associated with the biosynthesis of volatile fatty acid derivatives (e.g., methyl jasmonate), including lipoxygenase (*LOX*), alcohol dehydrogenase (*AHD*) and salicylate/benzoate carboxyl methyltransferase (*BSMT*) were influenced by both single and combined stress (Supplementary Table 10). All the *LOXs* were significantly down-regulated in CT while two *LOX1* were induced under CD but was induced to 7-folds upon DT. *ADH1/2/3* were over-represented in CD, while gene encoding *BSMT1* was down-regulated under all stress conditions. Gene encoding CCD enzyme that contributes to the formation of carotenoid-derived volatiles was found to be down-regulated in CD. Another *CCD* gene, *CCD4* showed up-regulation in CT, whilst it was down-regulated in DT. The majority of enzymatic genes involved in the biosynthesis of volatile terpenoids, such as *TES* and *TPD5/10/13*, were specifically induced by CT. Regarding the enzymes involved in the hydrolysis of glycosidically-bound volatile compounds, genes encoding beta-glucosidases 6/14/18/41/42 (*BGLU6/14/18/41/42*) were over-represented in DT. Phenylalanine ammonia-lyase (*PAL*) genes, which were related to the biosynthesis of volatile phenylpropanoids/benzenoids showed increased expression under all stress conditions.

## Discussion

**Figure 8:**
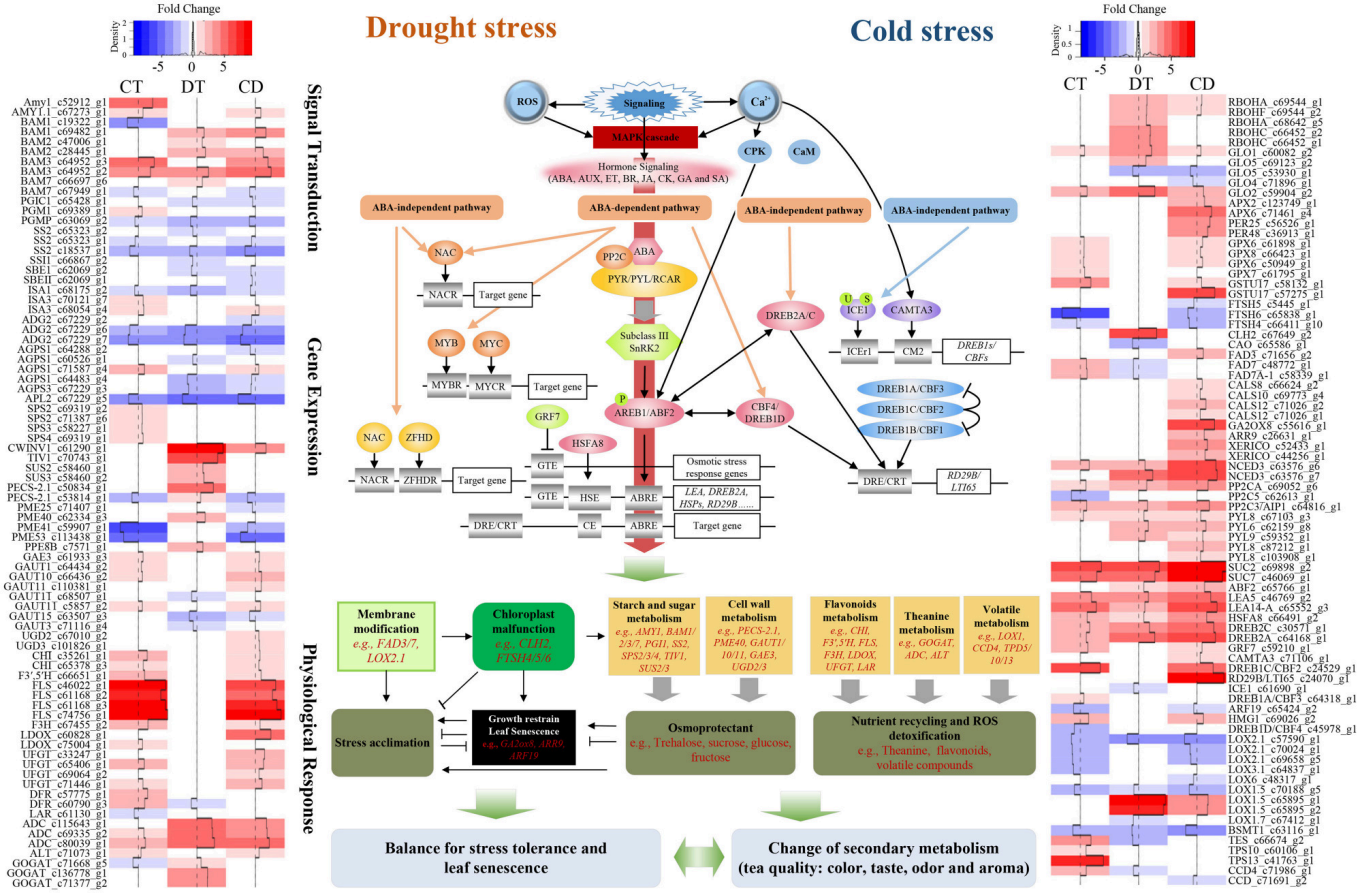
**Model representing the complex relationship between tea quality, leaf senescence and the responses to combined CD stress in *C. sinensis***. Sharp and blunt arrow denote activation and suppression, respectively. Heat maps show fold change of DEGs among the model during CT, DT, and CD. Full names of various genes are expanded in Supplementary Tables 11, 13.

Tea plants' responses to cold is well studied (Wang et al., 2013; Zheng et al., 2015). The modes of physiological and molecular responses to drought have been demonstrated in tea plants (Liu et al., 2015, 2016; Wang et al., 2016). However, those stressors might co-occur in the field, and cause leaf senescence as well as quality degradation; thus, marker-assisted breeding or cross-breeding, which targets single cold or drought stress, might be insufficient for improving the performance of tea plants in the field. Here we reported a comprehensive transcriptome study to characterize and compare the gene expression profiles of tea plants under cold, drought, and their combination. We also depict the complex relationship between stress responses, leaf senescence and tea quality (Figure 8; Supplementary Table 11). Our data will be closely relevant to agronomy and provide further insight into the basic questions about signaling cross-talk in systems biology (Mundy et al., 2006).

### The response to combined cold and drought stress does not reflect a simple merge of the single stress responses

We first compared the global transcriptome profiles of tea leaves under individual cold and drought stress with their combination. In the later, we further analyzed the transcriptional response behaviors under combined stress by clustering significantly responding unigenes from the CT, DT, and CD to predefined expression modes. We show that the interaction between cold and drought leads to both shared and unique response that ensures best utilization of limited energy resources of tea plants. Comparisons of DEGs across single and combined stress treatments have revealed that only 12.7% (2071) of genes were overlapped in all stress conditions, and only 47.4% (7721) of genes that activated by either CT or DT were able to respond to CD. Strikingly, the “canceled,” “prioritized,” and “combinatorial” response modes constitute 38% of the total unigenes, whose response patterns cannot be inferred from CT or DT experiments alone. Hence, the attempts to delineate signaling cross-talk under CD by simply identifying overlapping gene sets that are modulated by both CT and DT may be misleading, because over one-third of unigene response cannot be inferred from CT, or DT single-case studies. Additionally, unpredictable are unigenes that respond only to the CD and not to either CT and DT (3543, 31.5% DEGs of CD). Those CD-unique DEGs associated with redox (e.g., *GRXS10, PNC2, APX2*/*6, and PER25/48*), phytohormones balance (e.g., *IAA30, GA2OX8, PYL8, ARR9*, and *ERF061*/*118*), defense signaling (e.g., *CCAMK, CML31*, and *MPK9*/*19/20*), transcription regulation (e.g., *C3H, NAC002, MYB108, WRKY71, BHLH35*, and *VIP1*) and cell wall remodeling (e.g., *CALS8/1012, UPTG2, KAM1, GMGT1*, and *EXPA1*) may be important for evoking novel gene networks during CD and will be absent in analyses using only CT or DT experiments. Moreover, the CD-unique responses were composed of genes involved in pentose phosphate pathway, oxidative phosphorylation, fatty acid metabolism, fructose and mannose metabolism, galactose metabolism, alanine, aspartate, and glutamate metabolism, terpenoids metabolism, zeatin, folate, flavone, and flavonol biosynthesis, and various antioxidative pathways (e.g., peroxisome and glutathione) that contribute to inducible stress responses like membrane protection, ROS detoxification, theanine, flavone, and other secondary metabolites production. Recent omic studies have also demonstrated that there are unique hormone-responsive genes, TFs, osmolytes and secondary metabolites that are differentially modulated in response to different combined stresses (Atkinson et al., 2011; Rasmussen et al., 2013; Pandey et al., 2015; Gupta et al., 2016). Moreover, genes relevant to “growth” and “cell growth” were specifically up-regulated during CD, while “death” and “cell death” related genes were repressed. These results imply the sustaining of plant growth while negotiating with the stress response during combined occurrence of cold and drought.

Tea plants also shared a large number of unigenes to exhibit very concordant responses under cold and drought stress. Transcriptional response mode analysis revealed the existence of 26% of shared DEGs with “similar” expression pattern between individual and combined stress while only ~0% of DEGs exhibited “prioritized” mode upon combined CD stress. This is quite similar to the study of combined cold and high light stress, a common stress situation in temperate regions, which showed the highest level of “similar” mode (18.6%) and lowest level of “prioritized” mode (3.0%) transcripts among six pairs of combined stress (Rasmussen et al., 2013). This similarity is to be expected, since the common feature of both cold and drought is the promotion of cellular dehydration, and cold stress induces several responses similar to those changes observed in woody plants under drought stress (Yamaguchi-Shinozaki and Shinozaki, 2006; Beck et al., 2007). These shared responses including growth restrain, osmotic adjustment, ROS detoxification, activation of calcium-, phytohormone-, and MAPK-signaling pathway, as well as transcription regulation, which are general physiological and molecular adaptation of plants and can guard them against multiple single stresses (Figure 5). We also observed the different isoforms of a gene or different members of a gene family (based on gene IDs and gene descriptions) within several shared pathways (e.g., calcium signaling and MAPK signaling pathway) were differentially regulated between each stress treatment. This indicated that tea plants also elicit different isoforms or family members of these shared DEGs during single and combined stresses and likely opt the most efficient route to combat them.

During a combined stress situation, plants may be able to induce only the most effective genes to cope with the challenging situation with limited resources (Sewelam et al., 2014). Based on this hypothesis, we identified 319 genes from the 725 co-induced genes, whose expressions were tailored in response to CD and exhibited more enhanced expression compared to the individual stresses. Most of these shared genes have been known to impart abiotic stress resistance to plants and regulate plant growth via ABA-dependent and ABA-independent pathways. For instance, tea plants enhance the expression level of candidate genes, *DREB2s* (i.e., *DREB2A/C*), to cope with combined stress, although they cause some adverse effects to plant growth (Sakuma et al., 2006; Qin et al., 2007; Lee et al., 2010; Kim et al., 2012; Liu et al., 2013). Cold-induced *DREB1s/CBFs* (i.e., *DREB1A/C*), on the other hand, seem to improve the stress tolerance, and regulate the onset of drought-induced leaf senescence via its downstream genes, such as *RD29B/LTI65* (Yang et al., 2011; Koyama, 2014). It is likely that the strict control of different members of DREB family balance the trade-off between leaf growth and combined stress responses. Noteworthy, *GRF7* and *CAMTA3* which encode the inhibitor of DREB2A and the activator of DREB1C/CBF2, respectively, were significantly induced by CD and/or CT. The further activation of DREB1s/CBFs during cold acclimation may confers enhanced tolerance to drought stress, thereby leading to a prolonged life span in CD stressed plants. Hence, the manipulation of these candidate genes or their regulators, will be an effective strategy for the production of plant varieties resistant to concurrent cold and drought stress.

### Low temperature delays drought-induced leaf senescence and influences the accumulation of secondary metabolites in tea plants

The tea infusion color and shade of color in made tea are two attributes in the evaluation of various kinds of tea. The color in the infused leaf is largely determined by the chlorophyll, carotenoid and anthocyanin content, as well as the oxidation of flavonoids (e.g., catechin and gallocatechin). The color of the tea infusion is mainly influenced by water soluble anthocyanins, water soluble flavonols (e.g., myricetin, kaempferol, and quercetin), flavones (e.g., vitexin and isovitexin) and their glycosides, as well as the oxidation product of flavonoids (e.g., epicatechin gallate and epigallocatechin gallate) (Chaturvedula and Prakash, 2011). Furthermore, a delicious cup of tea infusion is believed to be an ingenious balance of various taste sensations, including sweetness, sourness, astringency, umami, and bitterness. Among these, the astringent taste was mainly determined by the contents of flavonoids, such as flavonols, catechins, and gallocatechin (Narukawa et al., 2010). During stress and drought-induced senescence, the degradation of chlorophyll along with the biosynthesis of carotenoid and anthocyanin unmasks the presence of carotenoids and anthocyanins results in the leaf yellowing and reddening. The stress-induced impairment of photosynthesis can lead to production of ROS, which is among the earliest events after initiation of systemic stress responses that might end up in the promotion of leaf senescence (Khanna-Chopra, 2012; Suzuki et al., 2012). Interestingly, the observed lower accumulation of ROS and/or higher antioxidant capacity seems to be a common mechanism operative in plants tolerant to different combined stresses (Sales et al., 2013; Pandey et al., 2015). In sugarcane plants, the drought-resistant genotype could maintain plant growth under cold and drought stress either alone or combined through enhancing the antioxidant capacity, which was not observed in the sensitive genotype (Sales et al., 2013). In the study on *A. mongolicus* subjected to individual cold and drought stress, genes involved in the flavonoid biosynthesis pathway were highly induced to cope with stress-induced oxidative damage (Wu et al., 2014). Both flavonoids and carotenoids belong to non-enzymatic antioxidants, which not only defend plants from oxidative damage but also show medicinal characteristics especially against human health problems (Nijveldt et al., 2001; Williams et al., 2004; Fiedor and Burda, 2014; Dani et al., 2016). In our studies, flavonoid and carotenoid biosynthetic pathways were significantly induced under all stress conditions, which seems to be important while protecting plant leaf cells from stress-induced oxidative damage. The production of flavonoids may depend on the nature and severity of stresses. CT and CD possibly contribute to the formation of more types of flavonoids, such as kaempferol, quercetin, and anthocyanidin due to the up-regulation of several key enzymatic genes (i.e., *CHI, F3*′,*5*′*H, F3H, FLS, LDOX*, and *UFGT*). In addition, CT may play a positive role in anthocyanidin accumulation but negatively influence the biosynthesis of catechin and gallocatechin through up-regulation of *DFR* and *LDOX*, and down-regulation of *LAR*. Altogether, the lifespan of tea leaves during combined CD stress may be extended by reducing the rate of ROS production and accumulating higher level of antioxidants (e.g., flavonoids and carotenoids).

Protein degradation occurs via the ubiquitin-proteasome system and through the action of proteases during abiotic stress and leaf senescence (Stone, 2014; Schippers et al., 2015). Chlorophyll disassembly allows the remobilisation of as much as 75% of the total cellular nitrogen present in stress-induced senescence leaves (Hörtensteiner and Feller, 2002). In addition, proteins can also be metabolized as alternative respiratory substrates when undergoing carbohydrate limitation during stress (Araújo et al., 2011). Theanine (g-glutamyl-L-ethylamide), an exclusive secondary metabolite, is one of the most abundant free amino acids found in tea leaves (it accounts for about 60–70% of total amino acids); and also distributed in all parts of young tea seedlings (Chen et al., 2012). This compound was known to impose tea infusion a unique umami taste (Yamaguchi and Ninomiya, 2000). Theanine biosynthesis in tea plants had shown to be influenced by drought and cold stress (Li et al., 2011; Wang et al., 2016). By activating the theanine synthesis pathway, the excess ammonium can be stored in theanine and tea plants can avoid damage from the high concentration of ammonium under abiotic stress (Li et al., 2011). Drought or drought-induced leaf senescence may facilitate the theanine biosynthesis in tea plants via up-regulating *ADC* and *GOGAT* to promote the accumulation of substrate ethylamine and glutamate, respectively. These theanine can be transported from senescing and/or older leaves to younger, growing tissues to support protein synthesis and N storage, which ensure the energy and metabolism homeostasis and contribute to plant survival during stress. On the other hands, however, the theanine biosynthesis seems to be negatively affected by delayed senescence under combined CD stress.

The sweetness taste of tea infusion was shown to be mainly contributed from various sugars, such as sucrose and glucose (Chaturvedula and Prakash, 2011). During stress, the degradation of starch takes place in tea leaves for supporting respiration under low photosynthesis condition and to produce osmolytes (glucose or other sugars) as an active response against the osmotic stress (Yue et al., 2015; Liu et al., 2016). Moreover, transcriptome changes in DT led to cell wall disassembly, while the maintenance of cell wall integrity were observed in CT and CD. The disassembly and modification of the cell wall under DT not only leads to ending the life of a cell in senescing leaves, but breaks down polysaccharides to soluble sugars, such as glucose and sucrose (Tenhaken, 2014). The presence of sufficient sucrose can serve as the important energy source for the cells, and they act as an essential osmoprotectant to protect biomembranes and proteins against abiotic stress (Cao et al., 2014). In addition, sucrose synthesis related genes were exclusively induced during CT, while sucrose degradation-related genes were more activated during DT than during CT and CD. These observations suggested that the bulk degradation of sucrose into glucose and fructose may be a strategy employed by tea plants to double its osmotic contribution in response to severe drought stress as reported in other plant species (Vargas et al., 2007; Ruan et al., 2010). In addition, sucrose transport and turnover during stresses may be much more important and complicated than in normal conditions, for example, sufficient sucrose should transport from source (e.g., leaves) to sink (e.g., roots) promptly to serve as an osmolyte (Durand et al., 2016). Hence, the highly induced sucrose transporter genes (*SUC2/7*) should be important candidates to cope with multiple stress conditions. Previous reports have revealed that both stress and sugar accumulation can promote leaf senescence (Masclaux-Daubresse et al., 2007; Wingler et al., 2015). In the perennial plant *Arabis alpina*, sugar accumulates strongly in response to cold stress while it is correlated with the senescence dependent decline in chlorophyll at warm temperature (Wingler et al., 2015). The observation that senescence is delayed and not accelerated despite sugar accumulation in the CT and CD can be explained with cold acclimation releasing the sugar-dependent repression of photosynthetic genes (Strand, 1997). Moreover, a possible link between variation in sugar sensitivity and in the expression of cold responsive genes has been found in the Bay-0 × Sha recombinant-inbred line population, suggesting that sugar signaling, senescence, and stress are related (Masclaux-Daubresse et al., 2007). Together, it is logical to infer that tea plants adjust the osmotic potential by breaking down polysaccharides to soluble sugars and accumulating different type of sugars according to the severity and nature of stress. Furthermore, cold acclimation possibly plays crucial roles in maintaining cell wall and changing senescence-induced sugar effect/sensitivity in CD stressed plants, where cell wall integrity and sugar accumulation is important for protection of mesophyll cells against combined CD stress.

Volatile compounds are essential for tea aroma and odor (Yang et al., 2013). Plant volatile compound emissions can be affected by abiotic factors according to the stress dose and nature. In *Solanum lycopersicum*, the emission of the terpenoids, and the LOX pathway products are quantitatively relevant to the severity of heat and frost stress (Copolovici et al., 2012). Both severe cold and heat stress induce a major emissions of LOX pathway products, whilst essentially no emissions were observed under mild stress. In addition, the emissions of terpenoids increased gradually with the severity of stress (Copolovici et al., 2012). In our study, CT may play a positive role in the biosynthesis of volatile terpenoids, such as beta-ionone which is significantly contribute to the flavor of black tea (Kawakami and Kobayashi, 2001). Both terpene synthase genes (e.g., *TES, TPD5/10/13*) and carotenoid cleavage oxygenase gene (e.g., *CCD4*) that implicated in the biosynthesis of volatile terpenoids were specifically induced by CT. The volatile terpenoids in leaves had been suggested to play a ubiquitous role in energy consumption and protecting membranes against oxidative stress (Vickers et al., 2009; Jardine et al., 2015). Furthermore, the repression of CCD genes in DT and CD possibly reflect the important status of carotenoids for redox homeostasis under severe stress condition. In addition to CCDs, LOX enzymes contribute to the formation of carotenoid-derived volatiles (Kawakami and Kobayashi, 2001). LOXs were also known to play a key role in the formation of fatty acid-derived volatiles (Yang et al., 2013), and involved in metabolism and hydrolysis of the membrane lipid in senescing leaves (Lim et al., 2007). In our study, single and combined stress can activate LOX pathway differently. Two LOX1 genes were induced in CD, whose expression levels were enhanced more pronounced in DT, while expression pattern of all the LOXs genes were significantly down-regulated by CT. These results suggested that drought stress possibly trigger emissions of volatile products of LOX pathway (such as various C6 aldehydes, alcohols, and derivatives) associated with oxidative burst. On the other hand, cold acclimation possibly delays drought-induced leaf senescence by attenuating lipid degradation of biomembrane under combined CD stress.

## Conclusions

To conclude, comparison of transcription profiles across single and combined stresses (involving cold and drought stress) revealed operation of mechanisms that (i) permit tea to tolerate combined CD stress through expression of the most effective as well as some unique genes associated with stress tolerance, (ii) delay drought-induced leaf senescence by modulation of a large amount of *SAGs* and cold responsive genes (e.g., *DREB1s* and *GRF7*), enhancement of antioxidant capacity (e.g., via promoting biosynthesis of flavonoids), attenuation of lipid degradation (e.g., via regulating terpenoids and LOX pathway), maintenance of cell wall and photosynthetic system, and changing of senescence-induced sugar effect/sensitivity during CD, as well as (iii) regulate secondary metabolism (e.g., flavonoids, theanine and terpenoids biosynthesis) that significantly influence the quality of tea under stress conditions (Figure 8; Supplementary Table 11). Candidate genes involved in the combined CD stress response and senescence-inhibition deserve further systematic functional validation. Further metabonomics studies are needed to validate the effect of both single and combined stresses on tea quality. In addition, the outcome of stress combination on plants depends largely on factors like growth stage of plant, timing and severity of stresses, and plant resistance to any one of the single stresses (Pandey et al., 2015). Therefore, care should be taken when utilizing a set of stresses to try and maximize leaf longevity and tea quality.

## Author contributions

ZD conceived the idea. ZD and CZ designed the study. CZ, YW, and LZ performed the experiments. CZ analyzed the data with the input from YW. ZD, and CZ wrote the manuscript. CZ, ZD, YW, and LZ critically read the manuscript.

### Conflict of interest statement

The authors declare that the research was conducted in the absence of any commercial or financial relationships that could be construed as a potential conflict of interest.

## Supplementary material

Supplementary Figure 1 **Functional annotation and classfication of *C. sinensis* transcriptome**. The functional category distribution of 170,102 non-redundant unigenes in **(A)** GO and **(B)** KEGG databases.

Supplementary Figure 2 **qRT-PCR validation for 16 DEGs**. Sixteen DEGs showed similar expression patterns between qRT-PCR assay and RNA-Seq data. The potential discrepancies between qRT-PCR and RNA-Seq results were indicated as blue triangles. Error bars indicate standard error of the mean expression values from three biological replicates.

Supplementary Figure 3 **Changing cutoffs**. The transcriptional responses investigated using different unigene sets shows consistent profiles. Set1, 500 most significant unigenes; Set2, 1000 most significant unigenes; Set3, 2000 most significant unigenes.

Supplementary Figure 4 **GO classification for the CD-unique DEGs**. GO classification of CD-uniquely **(A)** up-regulated and **(B)** down-regulated genes.

Supplementary Table 1 **Quality of sequencing**.

Supplementary Table 2 **Length distribution of assembled transcripts and unigenes**.

Supplementary Table 3 **Summary for the annotation of unigenes**.

Supplementary Table 4 **List of unigenes among the 20 transcriptional response modes**.

Supplementary Table 5 **List of selected differentially expressed *SAGs* with known effect**.

Supplementary Table 6 **List of CD-unique DEGs**.

Supplementary Table 7 **KEGG pathway classification of CD-unique DEGs**.

Supplementary Table 8 **List of DEGs involved in ROS production, starch and sucrose metabolism**.

Supplementary Table 9 **List of candidate genes for enhancing plant resistance to combined CD stress**.

Supplementary Table 10 **List of DEGs involved in the volatile, flavonoid and theanine biosynthetic pathway**.

Supplementary Table 11 **List of DEGs in the regulatory model**.

Supplementary Table 12 **Sequences of primers used for the qRT-PCR assay**.

Supplementary Table 13 **Abbreviations list**.
