# Supplementary figures and images for: Global Transcriptional Analysis Reveals the Complex Relationship between Tea Quality, Leaf Senescence and the Responses to Cold-Drought Combined Stress in Camellia sinensis
Source: Front Plant Sci. 2016 Dec 9;7:1858. doi: 10.3389/fpls.2016.01858 (PMC5145883; doi:10.3389/fpls.2016.01858)

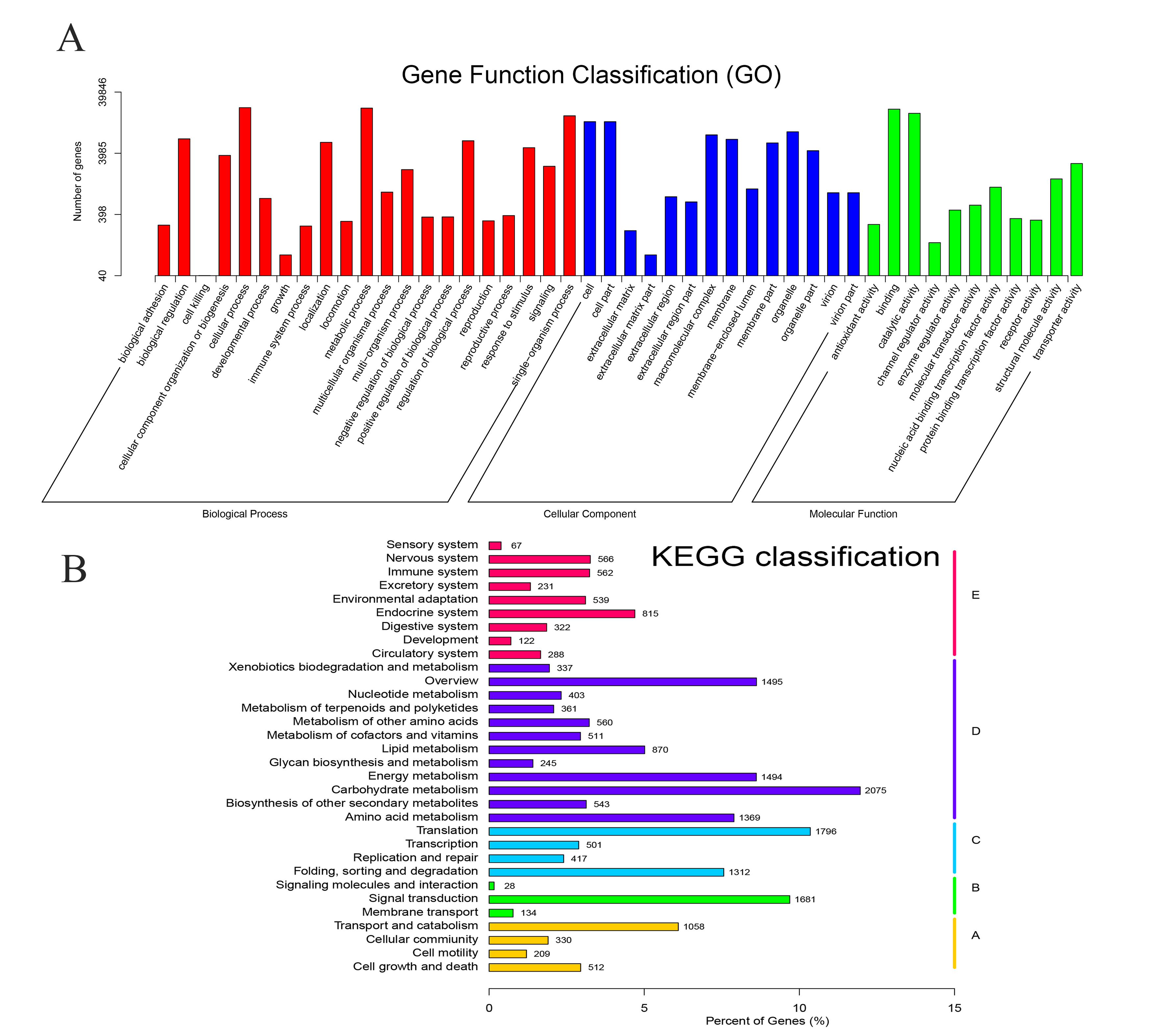

Supplement: Supplementary Figure 1 — Functional annotation and classfication of C. sinensis transcriptome. The functional category distribution of 170,102 non-redundant unigenes in (A) GO and (B) KEGG databases. [file Image1.TIF]

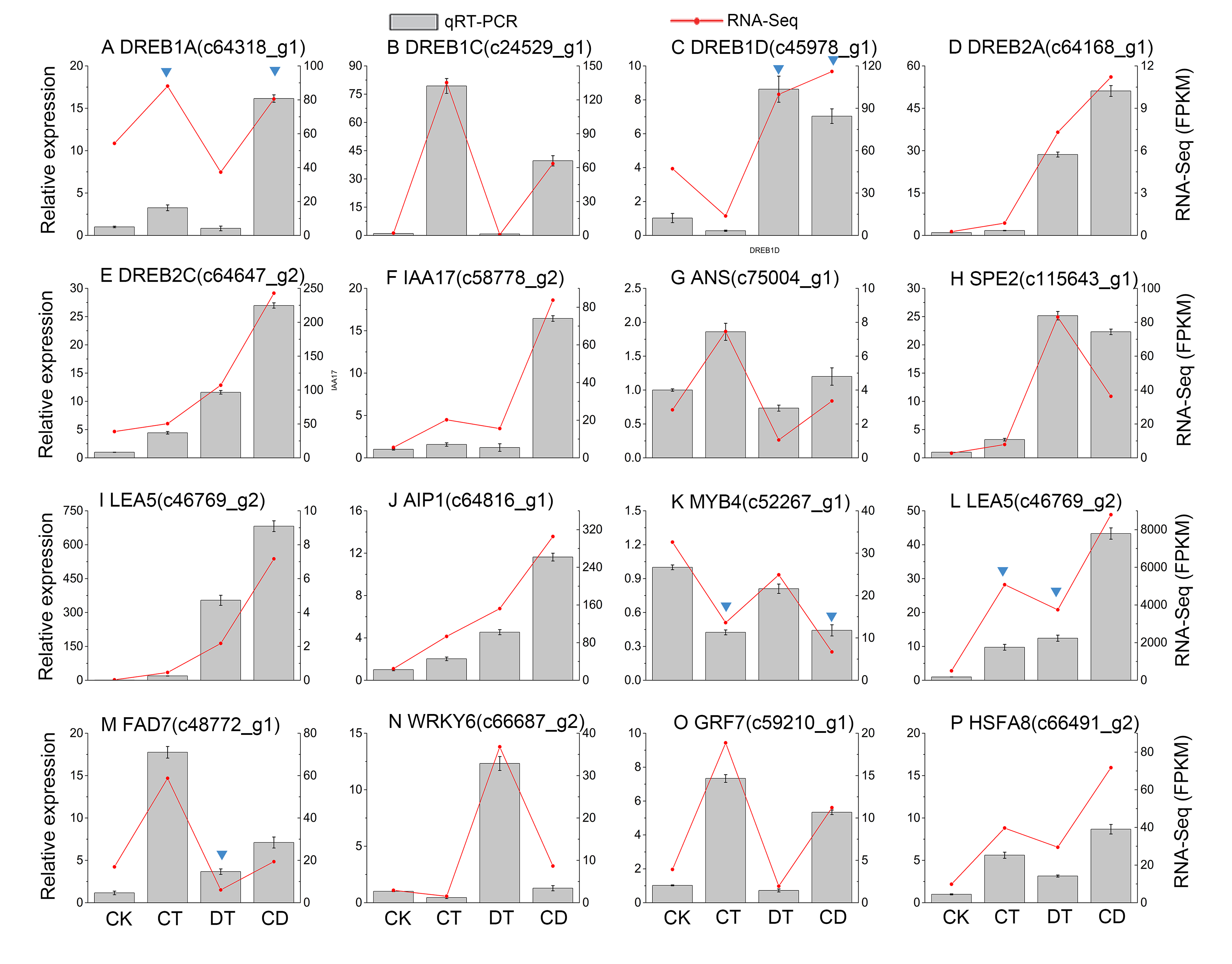

Supplement: Supplementary Figure 2 — qRT-PCR validation for 16 DEGs. Sixteen DEGs showed similar expression patterns between qRT-PCR assay and RNA-Seq data. The potential discrepancies between qRT-PCR and RNA-Seq results were indicated as blue triangles. Error bars indicate standard error of the mean expression values from three biological replicates. [file Image2.TIF]

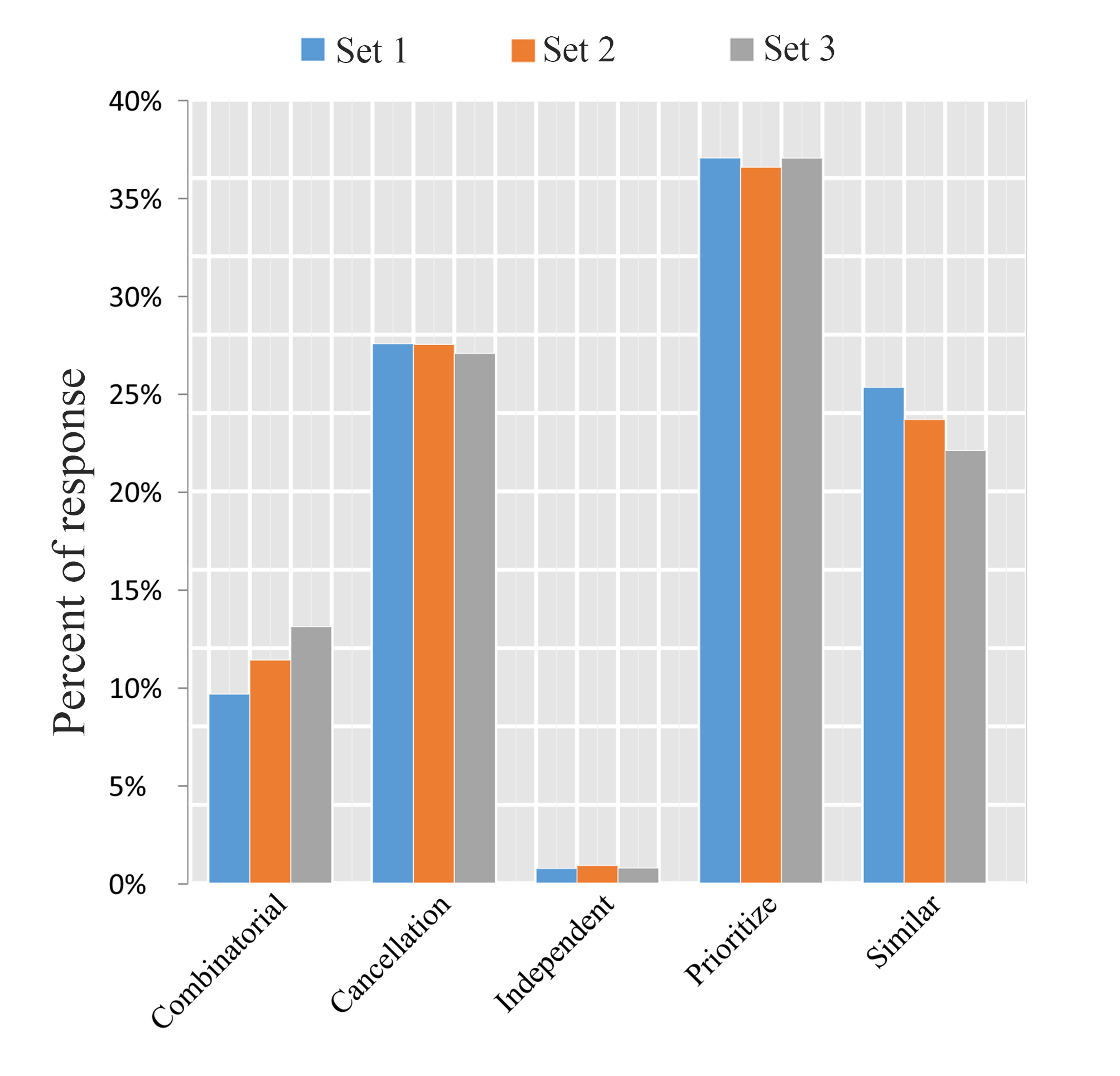

Supplement: Supplementary Figure 3 — Changing cutoffs. The transcriptional responses investigated using different unigene sets shows consistent profiles. Set1, 500 most significant unigenes; Set2, 1000 most significant unigenes; Set3, 2000 most significant unigenes. [file Image3.TIF]

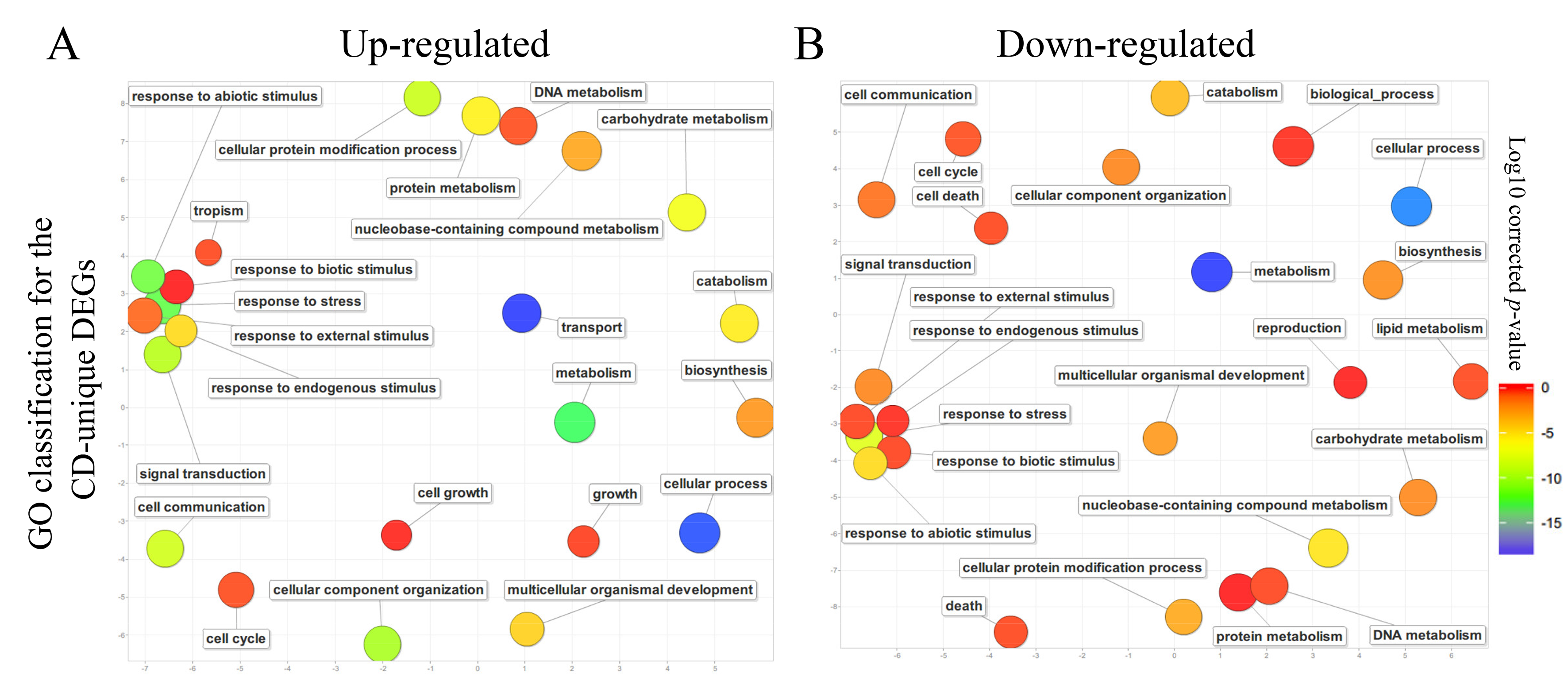

Supplement: Supplementary Figure 4 — GO classification for the CD-unique DEGs. GO classification of CD-uniquely (A) up-regulated and (B) down-regulated genes. [file Image4.TIF]
