# Supplementary material for: Global Transcriptional Analysis Reveals the Complex Relationship between Tea Quality, Leaf Senescence and the Responses to Cold-Drought Combined Stress in Camellia sinensis
Source: Front Plant Sci. 2016 Dec 9;7:1858. doi: 10.3389/fpls.2016.01858 (PMC5145883; doi:10.3389/fpls.2016.01858)
Supplement: Supplementary Table 1 — Quality of sequencing. [file Table1.DOCX]

**Supplementary Table 1. Quality of sequencing.**

| **Sample** | **Raw Reads** | **Clean reads** | **Clean bases** | **Error (%)** | **Q20 (%)** | **Q30 (%)** | **GC (%)** |
| --- | --- | --- | --- | --- | --- | --- | --- |
| CK1 | 49,937,178 | 47,082,346 | 7.06G | 0.02 | 95.64 | 89.72 | 46.66 |
| CK2 | 54,024,682 | 52,327,256 | 7.85G | 0.02 | 95.77 | 89.84 | 45.52 |
| CK3 | 44,130,872 | 42,376,870 | 6.36G | 0.02 | 94.73 | 87.6 | 46 |
| CT1 | 45,199,490 | 43,654,872 | 6.55G | 0.02 | 95.99 | 90.31 | 46.35 |
| CT2 | 40,203,194 | 39,101,684 | 5.87G | 0.02 | 95.87 | 90.03 | 45.67 |
| CT3 | 45,721,662 | 44,046,646 | 6.61G | 0.02 | 95.69 | 89.74 | 45.8 |
| DT1 | 50,903,512 | 49,100,574 | 7.37G | 0.02 | 96.02 | 90.39 | 45.85 |
| DT2 | 60,582,140 | 58,948,276 | 8.84G | 0.02 | 96.01 | 90.38 | 45.39 |
| DT3 | 50,364,530 | 48,186,232 | 7.23G | 0.02 | 96.36 | 91.01 | 45.35 |
| CD1 | 48,479,352 | 46,059,384 | 6.91G | 0.02 | 95.48 | 89.5 | 46.25 |
| CD2 | 44,982,944 | 43,827,354 | 6.57G | 0.03 | 94 | 86.08 | 45.75 |
| CD3 | 49,979,582 | 47,981,390 | 7.2G | 0.02 | 95.46 | 89.37 | 46.26 |
