# Supplementary material for: Global Transcriptional Analysis Reveals the Complex Relationship between Tea Quality, Leaf Senescence and the Responses to Cold-Drought Combined Stress in Camellia sinensis
Source: Front Plant Sci. 2016 Dec 9;7:1858. doi: 10.3389/fpls.2016.01858 (PMC5145883; doi:10.3389/fpls.2016.01858)
Supplement: Supplementary Table 2 — Length distribution of assembled transcripts and unigenes. [file Table2.DOCX]

**Supplementary Table 2. Length distribution of assembled transcripts and unigenes.**

| **Transcript length interval** | **Number of transcripts** | **Number of unigenes** |
| --- | --- | --- |
| **200-500bp** | 140,070 | 114,255 |
| **500-1kbp** | 55,691 | 31,463 |
| **1k-2kbp** | 43,635 | 15,389 |
| **>2kbp** | 29,493 | 8,995 |
| **Total** | 268,889 | 170,102 |
| **Min Length** | 201 | 201 |
| **Mean Length** | 871 | 615 |
| **Median Length** | 473 | 349 |
| **Max Length** | 14,576 | 14,576 |
| **N50** | 1,540 | 894 |
