# Supplementary material for: Global Transcriptional Analysis Reveals the Complex Relationship between Tea Quality, Leaf Senescence and the Responses to Cold-Drought Combined Stress in Camellia sinensis
Source: Front Plant Sci. 2016 Dec 9;7:1858. doi: 10.3389/fpls.2016.01858 (PMC5145883; doi:10.3389/fpls.2016.01858)
Supplement: Supplementary Table 3 — Summary for the annotation of unigenes. [file Table3.DOCX]

**Supplementary Table 3. Summary for the annotation of unigenes.**

|  | **Number of Unigenes** | **Percentage (%)** |
| --- | --- | --- |
| **Annotated in NR** | 53,292 | 31.32 |
| **Annotated in NT** | 33,452 | 19.66 |
| **Annotated in KO** | 17,348 | 10.19 |
| **Annotated in Swiss-Prot** | 36,399 | 21.39 |
| **Annotated in PFAM** | 38,299 | 22.51 |
| **Annotated in GO** | 39,846 | 23.42 |
| **Annotated in KOG** | 17,880 | 10.51 |
| **Annotated in all Databases** | 7,700 | 4.52 |
| **Annotated in at least one Database** | 66,433 | 39.05 |
| **Total Unigenes** | 170,102 | 100 |
